# An efficient ORF selection system for DNA fragment libraries based on split beta-lactamase complementation

**DOI:** 10.1371/journal.pone.0235853

**Published:** 2020-07-23

**Authors:** Vaishali Verma, Gopal Joshi, Amita Gupta, Vijay K. Chaudhary

**Affiliations:** 1 Centre for Innovation in Infectious Disease Research, Education and Training (CIIDRET), University of Delhi South Campus, New Delhi, India; 2 Department of Biochemistry, University of Delhi South Campus, New Delhi, India; Imperial College London, UNITED KINGDOM

## Abstract

PCR-based amplification of annotated genes has allowed construction of expression clones at genome-scale using classical and recombination-based cloning technologies. However, genome-scale expression and purification of proteins for down-stream applications is often limited by challenges such as poor expression, low solubility, large size of multi-domain proteins, etc. Alternatively, DNA fragment libraries in expression vectors can serve as the source of protein fragments with each fragment encompassing a function of its whole protein counterpart. However, the random DNA fragmentation and cloning result in only 1 out of 18 clones being in the correct open-reading frame (ORF), thus, reducing the overall efficiency of the system. This necessitates the selection of correct ORF before expressing the protein fragments. This paper describes a highly efficient ORF selection system for DNA fragment libraries, which is based on split beta-lactamase protein fragment complementation. The system has been designed to allow seamless transfer of selected DNA fragment libraries into any downstream vector systems using a restriction enzyme-free cloning strategy. The strategy has been applied for the selection of ORF using model constructs to show near 100% selection of the clone encoding correct ORF. The system has been further validated by construction of an ORF-selected DNA fragment library of 30 genes of *M*. *tuberculosis*. Further, we have successfully demonstrated the cytosolic expression of ORF-selected protein fragments in *E*. *coli*.

## Introduction

The availability of all the proteins encoded by the genome of any organism as purified preparations can facilitate numerous applications. These include functional annotation of genes identified during high-throughput genome sequencing projects, preparation of protein microarrays to study gene expression at different time-points or to determine the serological profiles of patients infected with a disease to identify immunodominant epitopes, isolation of specific antibodies to every protein encoded by a genome, etc. [1–6]. Such a resource can be produced by the amplification and cloning of all the Open Reading Frames (ORFs) encoded by an organism into appropriate vector systems, followed by their expression and purification [1, 7]. However, overexpression and purification of all the proteins encoded by a genome may not be a viable approach, especially while dealing with large complex genomes. Not all the proteins may express in a single heterologous host due to protein insolubility, and the problem is especially grave during expression of large multi-domain proteins, which are more prone to aggregation and may require a native milieu for their expression that may be often difficult to achieve [6, 8]. The yield and quality of proteins may also be compromised [8, 9]. In addition, this approach is time consuming and may not be very economical.

Alternatively, this feat can be accomplished by using DNA fragment libraries that can serve as the representatives of the protein complement of a genome either as DNA fragments encoding soluble overlapping protein fragments or as phage-displayed repertoires [1, 10]. DNA fragment libraries have been successfully employed for a variety of applications including mining of gut microbiome to study protein-protein interactions and identify novel biomarkers, epitope mapping of monoclonal and polyclonal antibodies, identification of soluble portions of multi-domain proteins, identification of immunodominant epitopes of pathogenic proteins using patient sera, etc. [10–19]. Such libraries are created by random fragmentation of the DNA encoding the target genome or selected gene sequences, followed by cloning into suitable expression or surface display vectors. However, due to the random DNA fragmentation, the libraries often carry a large number of non-functional fragments that encode stop codons due to frame shifts or incorrect orientation after cloning [only 1 out of 18 clones is useful; [20]]. Due to such a high proportion of non-functional clones, the downstream use of DNA fragment libraries becomes less efficient. Thus, there is a need to eliminate the DNA fragments encoding non-functional clones and enrich clones carrying the in-frame ORF.

Numerous systems have been reported in the literature to enrich libraries for in-frame clones. Gupta et al., employed a simple Fd-tet phage-based system for the construction of ORF-selected gene fragment libraries, where only the phages encoding DNA fragment in-frame with g3 were infectious and could be selected by infection in *E*. *coli* [21]. Similarly, Rondot et al., described a phage-packaging system comprising of a g3-deficient Hyperphage, in which the only source of g3p is the fusion protein encoded by the phagemid DNA and hence only the clones carrying in-frame fusion-g3p protein can produce infectious phage particles [22, 23]. Gupta et al., have also described a helper phage-AGM13, which carries trypsin-sensitive sites in g3, due to which the phages encoding in-frame ORFs can be selected by trypsin-treatment of the rescued phage population, followed by infection in *E*. *coli* [24]. However, all these systems involve multiple steps of library rescue, phage purification, re-infection, etc. Several groups have also described ORF selection systems based on different reporter genes. The enzyme Beta-lactamase has been widely used a reporter for selection of ORFs [1, 20, 25–28]. In these systems, the fragments are cloned between signal sequence and beta-lactamase and only in-frame fragments allow translation of full-length beta-lactamase and survival on ampicillin. Split-Dihydrofolate reductase has also been used for ORF selection of gene-fragment libraries [19].

This paper describes a highly efficient ORF selection system for DNA fragment libraries, which is based on a split beta-lactamase based protein fragment complementation. The sequence of split beta-lactamase in the ORF selection vector is based on the work described previously in the literature [29–32]. The system has been designed to allow seamless transfer of selected DNA fragment libraries into any downstream vector systems using a restriction enzyme-free cloning strategy. It has been optimized for the selection of ORFs using model constructs and has been validated by the construction of an ORF-selected DNA fragment library of 30 *M*. *tuberculosis* genes.

## Materials

*E*. *coli* strains TOP10F’ F' [*lacI*^*q*^ Tn10 *(tet*^*R*^)] *mcrA* Δ(*mrr-hsd*RMS*-mcr*BC) *φ80lacZΔM15 ΔlacX74 deoR nupG recA1 araD139 Δ(ara-leu)7697 galU galK rpsL(Str*^*R*^*) endA1 λ*^*-*^ and BL21 (DE3) RIL [B F^−^ *ompT hsdS* (rB^−^mB^−^) *dcm*+ Tet^r^ *gal l* (DE3) *endA Hte [argU ileY leuW* Cam^r^] were obtained from commercial sources. Genomic DNA from *M*. *tuberculosis* H37Rv was obtained as a gift from Prof. Anil. K. Tyagi, Department of Biochemistry, University of Delhi South Campus, New Delhi. The concentration of the *M*. *tuberculosis* H37Rv genomic DNA was ~ 1 μg/μl. Approximately 10 kb fragments obtained with mild sonication of the genomic DNA were used for the amplification of DNA encoding 30 *M*. *tuberculosis* H37Rv genes for construction of MTBLIB42 gene fragment library. The vector pJQ200SK was used as a template for amplification of *B*. *subtilis* SacB gene [33]. Oligonucleotides for cloning and NGS were obtained from Sigma-Aldrich, Bangalore, India and IBA Life sciences, Germany. Restriction enzymes and other DNA modifying enzymes were obtained from NEB, Ipswich, MA, USA. PfuUltra II Fusion HS DNA polymerase was obtained from Agilent Technologies, Santa Clara, US. Reagents for NGS were obtained from Illumina, Singapore. SureSpin plasmid miniprep kit was obtained from Genetix Biotech Asia Pvt. Ltd. and Qiagen QIAquick PCR or gel extraction kits were obtained from Qiagen, Hilden, Germany. Expand High Fidelity PCR system, dNTPs, and BSA was obtained from Roche, Mannheim, Germany. Mouse monoclonal antibody BA09-3 against Bla-Omega fragment (196–286 amino acids) was available in-house. PopCulture reagent, rLysozyme and Benzonase were from Merck, Germany.

## Methods

### Construction of ORF selection vector pVMAKORF001

Vector pVMAKORF001 is an arabinose-inducible pBAD promoter-based vector, which allows periplasmic expression of split beta-lactamase fusion proteins. The vector encodes an expression cassette comprising native beta-lactamase signal sequence (1–23 amino acids of TEM-1 beta-lactamase), Bla-Alpha fragment (24–195 amino acids of TEM-1 beta-lactamase) with M182T mutation [34], a tri-peptide Asparagine-Glycine-Arginine (NGR) after 195^th^ residue [30], SacR-SacB cassette encoding the *Bacillus subtilis* derived *levansucrase* gene flanked on either ends by two appropriately oriented BsaI sites and 15 amino acid glycine-serine rich spacers (G_4_S_3_) followed by Bla-Omega fragment (196–286 amino acids). The backbone comprises of T7 terminator, Fori, kanamycin resistance gene and ColE1 ori with deletion of *rop* gene. The vector was constructed by assembly of different cassettes using restriction enzymes. For cloning DNA between Bla-Alpha and Bla-Omega fragments, the vector uses a restriction enzyme-free cloning strategy as described before [35].

### Construction of model vectors for characterization of ORF selection system

Three vectors were constructed, namely, pVMAKORF19kDa001 (*M*. *tuberculosis* 19 kDa gene between Bla-Alpha and Bla-Omega; encoded protein referred as Alpha-19kDa-Omega), pVMAKORFWOI-In001 (WOI-Without Insert-In-frame; encoding in-frame spacer between Bla-Alpha and Bla-Omega; encoded protein referred as Alpha-Spacer-Omega) and pVMAKORFWOI-Off001 (encoding stop codon between Bla-Alpha and Bla-Omega; encoded protein referred as Alpha-Stop).

To obtain pVMAKORF19kDa001, the gene encoding *M*. *tuberculosis* 19 kDa protein (Rv3763) was amplified using gene-specific primers, which added a DNA sequence encoding Tobacco Etch Virus (TEV) protease site followed by PmeI site at the 5’ end, and SwaI site followed by glycine-serine rich spacer sequence at the 3’ end (because similar adapter sequences were appended to the fragment during the construction of DNA fragment libraries) and were compatible with restriction enzyme-free cloning strategy [35]. The PCR product was processed using Column method and was cloned in BsaI-digested pVMAKORF001 vector as described before [35, 36]. The vector was transformed in *E*. *coli* TOP10F’ cells.

To obtain pVMAKORFWOI-In001 or pVMAKORFWOI-Off001, the NheI-BssHII digested backbone of pVMAKORF001 was treated with T4 DNA polymerase in the presence of dNTPs and subjected to self-ligation using T4 DNA ligase followed by electroporation in *E*. *coli* TOP10F’ cells. From our previous experience, it was known that the process of blunting and ligation might lead to deletion of 1–2 bases at the ligation junction in ~ 50% clones. Thus, by this self-ligation-based cloning, both types of clones, i.e. either carrying perfect in-frame sequence or carrying 1 base deletion (leading to frameshift and generation of an in-frame stop codon) at the ligation junction were obtained upon DNA sequence analysis.

### Western blot analysis of periplasm fraction of the model constructs

Three clones, namely pVMAKORF19kDa001, pVMAKORFWOI-In001 and pVMAKORFWOI-Off001 were grown in LB-Kan_30_ media (LB media containing 30 μg/ml kanamycin) at 30°C, 250 rpm up to OD_600nm_ ~0.8, and induced with 0.05% arabinose for 1 hr at 30°C. Periplasm was prepared using modified osmotic shock method as described before [37]. Two-fold dilutions of periplasm samples were resolved using SDS-PAGE under reducing conditions and proteins were transferred to 0.45 μ PVDF membrane. Western blot was performed with 1 μg/ml anti-Bla-Omega mouse monoclonal antibody BA09-3.

### Analysis of growth of model constructs at increasing concentrations of ampicillin

Three clones, namely pVMAKORF19kDa001, pVMAKORFWOI-In001 and pVMAKORFWOI-Off001 were grown in MDAG-Kan_30_ media (MDAG media [38] supplemented with 30 μg/ml kanamycin) at 30°C, 250 rpm till OD_600nm_ ~0.6. The cells were pelleted by centrifugation at 5000 rpm at RT and resuspended in MDA-Gly_0.5%_Kan_30_ media [MDAG media without glucose; named as MDA; supplemented with 0.5% glycerol as the carbon source]. The cultures were incubated for 30 min at 30°C, 250 rpm and then pre-induced with 0.0002% arabinose for 30 min at 30°C, 250 rpm. The induced cultures were diluted serially in MDA-Gly_0.5%_Kan_30_ media and spotted with 96-well replicator on plates containing LB-Kan_30_ media supplemented with 0.0002% arabinose and 0, 10, 50, and 100 μg/ml ampicillin followed by incubation at 37°C for 16 hr.

### Determination of the efficiency of ORF selection using model constructs

The in-frame clone pVMAKORF19kDa001 and the off-frame clone pVMAKORFWOI-Off001 were grown at 30°C, 250 rpm and pre-induced with 0.0002% arabinose as described above. Based on OD_600nm_, the pre-induced cultures of in-frame and off-frame clones were mixed in 1:20 ratio, respectively, and 10 fold dilutions were plated on 90 mm plates containing LB-Kan_30_ media supplemented with 0.0002% arabinose and 0 or 10 μg/ml ampicillin, followed by incubation at 37°C for 16 hr. Twenty-four and seventy-two colonies from the plates containing 0 μg/ml and 10 μg/ml ampicillin, respectively were analyzed by colony PCR as described before [35] using 5’ primer BlaAlpha51 (TTGCGCAAACTATTAACTGGCGA) and 3’ primer BlaOmega31 (ACTTTATCCGCCTCCATCCAGTC). PCR products were analyzed on 1.2% agarose gel, and representation of the ORF *versus* non-ORF clones was determined based on the amplicon size.

### Construction of *M*. *tuberculosis* H37Rv 30 gene fragment library in pVMAKORF001 vector

For the construction of *M*. *tuberculosis* 30 gene fragment library, the protocol described in [35] was further improved to obtain a superior quality library. Based on the literature, thirty *M*. *tuberculosis* H37Rv genes with immunodiagnostic potential were chosen for construction of the gene fragment library (S1 Table). Based on their size, the genes were divided into 3 groups, namely A, B, and C (S1 Table). The genes were classified as secretory or cytosolic based on signal sequence prediction using online available software SignalP 3.0 version [39]. However, based on the literature, ESAT-6 and CFP10 were designated as secretory proteins despite no signal sequence prediction [40]. For all the proteins predicted to have signal sequence, the primers were designed to amplify only the sequence encoding mature protein (after signal sequence removal), whereas, for the remaining genes (i.e. cytosolic), the primers were designed to amplify full-length genes. However, Rv0934 (38 kDa Ag), was amplified both as full-length gene and as gene encoding only mature protein, as some research groups employed full-length protein during immunodiagnostic assay development [41]. In addition, the 3’ primer for each gene was designed to exclude the stop codon at the 3’ end of the gene during amplification. The genes were amplified using *M*. *tuberculosis* H37Rv genomic DNA (available as ~10 kb fragments; obtained after mild sonication) as a template.

Each of the 17 genes in group A and 11 genes in group B were amplified in a reaction volume of 100 μl containing 200 μM dNTPs, 5 ng sonicated *M*. *tuberculosis* H37Rv genomic DNA fragments, 50 pmoles each of respective 5’ and 3’ primer (S2 Table), 2% DMSO, and 2.1 U High Fidelity DNA polymerase in 1 x High Fidelity PCR buffer for 30 cycles of PCR as per manufacturer’s instructions. The PCR products were purified using QIAquick spin PCR purification kit. Five genes namely, Rv1860 (MPT32), Rv2970c (LipN), Rv3763 (19 kDa Ag), Rv3841 (BfrB), and Rv3864 (EspE) were further purified from 1.2% Seakem GTG agarose gel stained with SYBR Safe DNA stain using QIAquick gel extraction kit as per manufacturer’s instructions. Group C genes were amplified using PfuUltra II Fusion HS DNA polymerase. Rv0538 (PTRP) was amplified in a 100 μl reaction containing 4% DMSO, 200 μM dNTPs, 50 pmoles each of 5’ primer Rv0538-51 and 3’ primer Rv0538-31, 5 ng sonicated *M*. *tuberculosis* H37Rv genomic DNA fragments and 2 U of DNA polymerase in 1 x PfuUltra II Fusion HS buffer for 30 cycles of PCR as per manufacturer’s instructions. The PCR product was purified using QIAquick spin PCR purification kit. Rv1837c (Malate synthase, 81 kDa Ag) gene was amplified using 2 step PCR. First step PCR was performed using primers annealing to the region immediately upstream and downstream of the Rv1837c gene coding sequence. PCR was set up in 100 μl reaction volume containing 4% DMSO, 200 μM dNTPs, 50 pmoles each of 5’ primer Mtb81-51 and 3’ primer Mtb81-31, 5 ng sonicated *M*. *tuberculosis* H37Rv genomic DNA fragments and 2 U of DNA polymerase in 1 x PfuUltra II Fusion HS buffer for 30 cycles of PCR as per manufacturer’s instructions. The PCR product was purified using QIAquick spin PCR purification kit. Second step PCR was performed using first step PCR product as a template with gene-specific primers for amplification of full-length Rv1837c gene. PCR was set up in 100 μl reaction volume containing 6% DMSO, 200 μM dNTPs, 50 pmoles each of 5’ primer Rv1837c-51 and 3’ primer Rv1837c-31, 5 ng of purified first step PCR product and 2 U of DNA polymerase in 1 x PfuUltra II Fusion HS buffer for 30 cycles of PCR as per manufacturer’s instructions and the PCR product was purified using QIAquick spin PCR purification kit. The purified PCR products of all the genes from group A, B, and C, were analyzed on 1.5% agarose gel.

Group A, B, and C genes were fragmented separately to obtain gene fragments in the size range of 150–400 bp. Since the group A genes were small (in the size range of 285–800 bp), twice the amount of group A genes was sheared. In addition, two genes namely Rv3874 (CFP10; 300 bp) and Rv3875 (ESAT-6; 285 bp) were not included in the shearing process because their size was already in the desired size range of 150–400 bp and were spiked as full-length genes in the sheared mix. For group A, 7 pmoles of each gene was pooled to yield pool A. For group B and C, 3.5 pmoles each of all the genes were pooled to yield pool B and pool C, respectively (S1 Table). The three pools were sheared individually using acoustics-based focused ultrasonicator (Covaris S220) in 100–1500 bp compatible Fiber snap-cap AFA microtubes containing approximately 5 μg DNA in a volume of 130 μl Qiagen EB (per run) at peak incident power (W) = 175, duty factor = 10% and 200 cycles per burst for 180 sec to obtain fragments in the size range of 100–500 bp. The sheared DNA from all three groups was pooled and analyzed on agarose gel. Remaining DNA was concentrated using ethanol-sodium acetate precipitation and subjected to agarose gel-based size selection on 1.2% Seakem GTG agarose gel stained with SYBR Safe DNA stain for visualization under blue light. A ~ 150–400 bp band was excised and DNA was purified using QIAquick gel extraction kit as per the manufacturer’s protocol. The fragments were quantified using Qubit Fluorometer 2.0 (Thermo Fisher Scientific, Waltham, USA) employing dsDNA BR kit as per the manufacturer’s protocol. Approximately, 10 μg of sheared DNA (from 28 genes) was mixed with 6 ng each of purified genes Rv3874 (CFP10) and Rv3875 (ESAT-6), to complete the representation of all 30 genes chosen for library construction. This mixture was employed for further processing required for the construction of library.

Approximately 10 μg DNA fragments in the size range of approximately 150–400 bp were subjected to end repair and 5’ phosphorylation using a mixture of T4 DNA polymerase and T4 Polynucleotide Kinase in the presence of dNTPs, using Quick blunting kit (New England Biolabs, Ipswich, USA) as per manufacturer’s protocol followed by purification using QIAquick PCR purification kit. The purified end-repaired DNA fragments were treated with Klenow DNA polymerase (3’ → 5’ exo-) in the presence of dATP. The A-tailed DNA fragments were ligated to 3’ T-tailed oligo adapter duplex encoding TEV protease site (Duplex L; 34 mer; with 5’ biotin) and Gly-Ser rich spacer (Duplex K; 31 mer) (S3 Table) using T4 DNA ligase. Following ligation, the unligated adapters and/or adapter dimers were separated from the adapter-ligated DNA fragments using AMPure XP beads (in 1.2 x v/v ratio; Beckman Coulter) and purified DNA fragments were eluted in 70 μl Qiagen EB. The purified adapter-ligated DNA fragments were subjected to nick-repair reaction to seal the nicks that remained after adapter ligation using Bst Polymerase large fragment in the presence of dNTPs as per manufacturer’s protocol, followed by purification of the reaction using QIAquick PCR purification kit. Finally, the fragments carrying different adapters on either ends (i.e. 5’-*Adapter L*-DNA Fragment-*Adapter K*-3’ or 5’-*Adapter K*-DNA Fragment-*Adapter L*-3’) were selected from the fragments containing same adapters on either ends (i.e. 5’-*Adapter L*-DNA Fragment-*Adapter L*-3’ or 5’-*Adapter K*-DNA Fragment-*Adapter K*-3’) and a single-stranded DNA library was prepared as described before [35]. The adapter-ligated ssDNA 30 gene fragment library was further amplified using emulsion PCR to obtain dsDNA fragment library. For this, 260 μl x 3 aqueous PCR were setup, each containing 0/1/2% DMSO along with 0.5 ng ssDNA library template (= 3 x 10^9^ molecules; Average size = 300 bp), 10 mg/ml BSA, 200 μM dNTPs, 78 pmoles each of 5’ primer L3-s and 3’ primer K2-s, and 6 U of PfuUltra II Fusion HS polymerase in 1 x PfuUltra II Fusion HS polymerase buffer. The aqueous PCR was emulsified as described before [42] and subjected to 35 cycles of PCR as per manufacturer’s instructions. The ePCR products were purified as described before [42]. The amplified dsDNA PCR products from all three reactions (0/1/2% DMSO) were pooled and subjected to size selection using agarose gel stained with SYBR Safe DNA stain to obtain fragments in the size range of 200–400 bp. The size-selected 30 gene DNA fragment library (~ 220–400 bp) was subjected to T4 DNA polymerase treatment in the presence of dTTP to generate 4 base overhangs at 5’ ends of the DNA fragments using library-scale method for insert preparation as described before [35]. The fragments were quantified using Qubit Fluorometer 2.0 (Thermo Fisher Scientific, Waltham, USA) employing dsDNA BR kit as per manufacturer’s protocol. The ligation reaction was set up with BsaI-HF digested vector pVMAKORF001 and T4 DNA Polymerase treated 220–400 bp DNA fragments to obtain a library of ~ 5–10 x 10^7^ transformants as described before [35]. The reaction mixture was electroporated in *E*. *coli* TOP10F’ electrocompetent cells (electroporation efficiency = 5 x 10^9^ transformants per μg supercoiled pGEM-3Z DNA) and approximately 8 x 10^7^ clones were obtained at an electroporation efficiency of 4 x 10^8^ per μg of ligated DNA. The cells were scraped in MDAG media, mixed with equal volume of glycerol storage solution, and stored as 1.0 ml aliquots at -80°C. This library comprising of ~ 8 x 10^7^ unselected primary transformants was called as MTBLIB42C01. Twenty-four clones were screened by colony PCR with 5' primer BlaAlpha51 and 3’ primer BlaOmega31 as described before [35]. Approximately 1 x 10^9^ cells from MTBLIB42C01 were inoculated in 20 ml MDAG-Kan_30_ media, and culture was grown at 37°C at 250 rpm. Plasmid DNA was purified from 3 ml culture using SureSpin plasmid miniprep kit.

### ORF selection of MTBLIB42C01 library

For analytical ORF selection, ~ 6 x 10^8^ cells from MTBLIB42C01 library were inoculated in 20 ml pre-warmed MDAG-Kan_30_ media. The culture was grown at 30°C, 250 rpm until OD_600nm_ ~ 0.6–0.7 was reached followed by centrifugation at 3000 rpm for 15 min at RT. The pellet was re-suspended in 20 ml MDA-Gly_0.5%_ media. The culture was grown at 30°C, 250 rpm for 30 min. For pre-induction, arabinose was added to the culture to a final concentration of 0.0002% and culture was induced at 30°C, 250 rpm for 30 min. Following induction, 10 fold serial dilutions of the culture were prepared in MDA-Gly_0.5%_Ara_0.0002%_ and 1 ml each of 10^1^−10^6^ dilutions was plated on 150 mm petriplates containing LBKan_30_Ara_0.0002%_Amp_20_ media (LB media supplemented with 30 μg/ml kanamycin, 0.0002% arabinose, and 20 μg/ml ampicillin). Simultaneously, an aliquot of the dilutions was plated on non-selective MDAG-Kan_30_ plates to determine the cell count (= 1.8 x 10^8^ cells/ml). The plates were incubated at 37°C for 16 hours. Sixteen colonies from 10^2^ (lawn) and 10^3^ dilution plates (8–10 x 10^3^ colonies) were streaked on MDAG-Kan_30_ (no ampicillin) to obtained well-isolated colonies, which were screened by colony PCR with 5’ BlaAlpha51 and 3’ BlaOmega31 primers and the PCR products were sequenced as described before [35]. The sequences obtained were aligned to *M*. *tuberculosis* genome using MacVector 12.5.1 to determine the number of in-frame clones.

Based on analytical ORF selection results, preparative scale ORF selection was performed to obtain an ORF-selected library of ~ 4 x 10^5^ clones. Approximately 6 x 10^8^ cells of MTBLIB42C01 were inoculated in 20 ml pre-warmed MDAG-Kan_30_ media. Culture and grown and pre-induced same as described above. Following induction, 10 fold serial dilutions of the culture were prepared in MDA-Gly_0.5%_Ara_0.0002%_ and 1 ml each of 10^3^ dilution (= 1.4 x 10^5^ cells based on cell count determined by plating dilution on non-selective MDAG-Kan_30_ plates) was plated on fifty 150 mm petriplates containing LBKan_30_Ara_0.0002%_Amp_20_. The plates were incubated at 37°C for 16 hours. Each plate carried approximately 1 x 10^4^ colonies, and the cells from fifty plates were scraped in 75 ml MDAG media, mixed with equal volume of glycerol storage solution, and stored as 1.0 ml aliquots at -80°C. A total library size of approximately ~ 4 x 10^5^ clones was obtained. This library comprising of ORF-selected clones was named as MTBLIB42C02. Approximately 2 x 10^8^ cells from MTBLIB42C02 were inoculated in 20 ml MDAG-Kan_30_ media, and culture was grown at 37°C for 16 hr at 250 rpm. Plasmid DNA was purified from 6 ml culture using SureSpin plasmid miniprep kit.

### Preparation of dual-indexed MTBLIB42C01 and MTBLIB42C02 libraries for next-generation sequencing

Dual-indexed MTBLIB42C01 and MTBLIB42C02 libraries were prepared by PCR amplification of cloned DNA fragments from MTBLIB42C01 and MTBLIB42C02 using in-house developed strategy comprising of two overlapping primers for addition of sequences required for NGS on MiSeq (Illumina Platform). Emulsion PCR was performed under conditions optimized above. For each library, aqueous PCR was set up in a volume of 390 μl containing 200 μM dNTPs, 10 mg/ml BSA, ~ 4.5 x 10^9^ molecules of template DNA (3 x 10^9^ molecules per 260 μl aqueous PCR), 117 pmoles each of 5’ overlapping primer mix (Inner:Outer primer at 1:10; index 502) and 3’ overlapping primer mix (Inner:Outer primer at 1:10; index 702), and 9 U PfuUltra II Fusion HS polymerase in 1 x PfuUltra II Fusion HS polymerase buffer. The PCR mix was divided into 3 equal aliquots and DMSO was added at final concentration of 0/1/2%. About 125 μl aqueous reaction was emulsified in 250 μl oil-surfactant mix as described above and emulsion was divided as 50 μl aliquots in 0.2 ml PCR tubes and overlaid with 30 μl mineral oil followed by PCR for 30 cycles. The ePCR mixture was pooled and purified using QIAquick PCR purification kit same as described above. The ePCR product was eluted in 60 μl EB and further purified and concentrated using Ampure XP beads (1.2 x v/v ratio) and an aliquot was analyzed on 1.5% agarose gel. The libraries were quantified using dsDNA HS quantification kit with Qubit Fluorometer (version 2.0).

### Next generation sequencing of MTBLIB42C01 and MTBLIB42C02 libraries using MiSeq sequencer (Illumina)

The PCR amplified dual-indexed MTBLIB42 libraries were loaded at 6 pM concentration further spiked with 15% PhiX high-diversity control library (cat no. FC-110-3001, Illumina). The libraries were diluted and denatured as per manufacturer’s instructions. PhiX control library was similarly denatured and diluted to 6 pM. To obtain the “final library mix” containing 6 pM MTBLIB42 library with 15% PhiX, 850 μl of 6 pM denatured library was mixed with 150 μl 6 pM denatured PhiX library and sequencing was performed using MiSeq Nano v2 reagent kit for 2 x 250 cycles of paired-end sequencing as per manufacturer’s instructions. The raw data is available at the Sequence Read Archive (NCBI) under BioProject accession number PRJNA639215.

The sequencing data was analyzed using an in-house developed pipeline (ORFSELECT) employing a combination of open-source tools, Perl and shell scripts to determine the number of in-frame reads in the MTBLIB42 library before (C01) and after (C02) ORF selection. The analysis was performed on a HP Z400 workstation with 16 GB RAM and 6 core processors in Centos 7 Linux environment. The open-source tools included gzip (version 1.5), SeqPrep, cutadapt (version 1.16) [43], bowtie2 (version 2.3.4.1) [44], GNU-AWK (version 4.0.2), transeq (EMBOSS; version 6.6.0) [45], NCBI BLAST tool (version 2.7.1) [46] and Perl (version 5.24.4). The pipeline requires six user-supplied information including (i) Fastq file of Read1, (ii) Fastq file of Read2, (iii) Nucleotide reference fasta file, (iv) Amino acid reference fasta file, (v) 3’ adapter sequence, and (vi) 5’ adapter sequence, and involves 10 steps that are executed sequentially. Briefly, the pipeline involves merging of paired-end sequence reads, trimming of adapters in merged sequences, mapping of reads to reference, conversion of sequence alignment map (SAM) file to binary alignment map (BAM) file, sorting of BAM file, extraction of coordinates of aligned reads in SAM format, conversion of SAM file into tabular format, conversion of tabular format to Fasta format, translation of DNA sequence to protein sequences, and selection of in-frame clones. The pipeline will be available upon request.

Simultaneously, the number of theoretically possible in-frame clones for 30 *M*. *tuberculosis* gene fragment libraries of size 100 bp, 200 bp and 300 bp was also calculated. For this, overlapping 30 *M*. *tuberculosis* gene-fragment libraries of 100 bp, 200 bp and 300 bp fragment size with 1 bp incremental shift (to mimic random DNA fragmentation) were generated *in silico* using Perl scripts and the dataset was analyzed using ORFSELECT pipeline.

### Transfer of MTBLIB42C02 into pVMH10D-BAP001 expression vector

Plasmid DNA purified from culture representing 2 x 10^8^ cells of MTBLIB42C02 library (size ~ 4 x 10^5^) was used as a template for the amplification of ORF-selected fragments. Three aqueous PCR were set up in a volume of 260 μl each containing 0%, 1% or 2% DMSO and 200 μM dNTPs, 3 x 10^9^ molecules of template DNA, 78 pmoles each of 5’ primer L3-s and 3’ primer K2-s, 6 U PfuUltra II Fusion HS polymerase in 1 x PfuUltra II Fusion HS polymerase buffer. Approximately 250 μl PCR was emulsified in 500 μl oil-surfactant mix, aliquoted in 0.2 ml PCR tubes, and 35 cycles of PCR were performed followed by purification of PCR product as described above. The purified fragments were quantified using dsDNA BR quantification kit with Qubit Fluorometer (version 2.0). Approximately 2 μg purified fragments were subjected to T4 DNA polymerase treatment in the presence of dTTP to generate 4 base overhangs at 5’ ends of the DNA fragments using library-scale method for insert preparation as described before [35]. The insert preparation was analyzed on agarose gel, quantified using dsDNA BR quantification kit with Qubit Fluorometer (version 2.0), and used for ligation in expression vector pVMH10D-BAP001. It is a T7 promoter-lac operator-based expression vector that allows cytosolic expression of recombinant protein fragments carrying N-terminal deca-histidine tag (H10), bacteriophage lambda D coat protein and C-terminal BAP tag. The N-terminal deca-histidine tag facilitates the purification of the expressed recombinant protein using affinity chromatography, and the bacteriophage lambda D coat protein serves as a solubility enhancer for protein fragments. The 15 amino acid C-terminal BAP tag is required for the site-specific biotinylation of protein fragments [36]. Ligation was performed in a reaction volume of 10 μl containing 100 ng BsaI-digested pVMH10D-BAP001 vector, T4 DNA polymerase-treated MTBLIB42C02 DNA fragments (3 molar excess) and 1 U of T4 DNA ligase (5 U/μl, Roche) in 1 x ligation buffer as described before [35]. The reaction mixture was electroporated in *E*. *coli* BL21 (DE3) RIL electrocompetent cells and regenerated cells were plated on MDAG-Amp_100_Cm_30_ plates. The colonies were scraped in MDAG media, mixed with equal volume of glycerol storage solution, and stored at -80°C. A total library size of ~3 x 10^6^ clones was obtained. Sixteen clones were screened by colony PCR as described before [35].

### Expression and subcellular localization of randomly selected clones of MTBLIB42C02 library in pVMH10D-BAP001 vector

Twelve clones encoding protein fragments from MTBLIB42C02 library in pVMH10D-BAP001 vector were selected for recombinant protein expression by auto-induction [38]. The clones were inoculated in 3 ml MDAG-Amp_100_Cm_30_ media and grown at 37°C, 250 rpm for 16 hours. The overnight grown culture was diluted 1:100 in 3 ml auto-induction media ZYM5052Amp_100_Cm_30_. The culture was grown with constant shaking at 250 rpm for 2 hr at 30°C, 4 hr at 24°C, followed by 16 hr at 18°C. To determine the solubility of the proteins, PopCulture reagent-based solubility assay was performed. For this, 50 μl of PopCulture reagent (containing rlysozyme and Benzonase) was added to 500 μl of induced culture and incubated on ice for 30 minutes. Total cell sample was saved, and remaining sample was centrifuged at 22,000*g* for 30 minutes at 4°C and supernatant (Soluble) was collected. The total cell extract and soluble protein samples were analyzed using SDS-PAGE under reducing conditions.

## Results

### Concept of ORF selection using split beta-lactamase protein fragment complementation system

**Fig 1:**
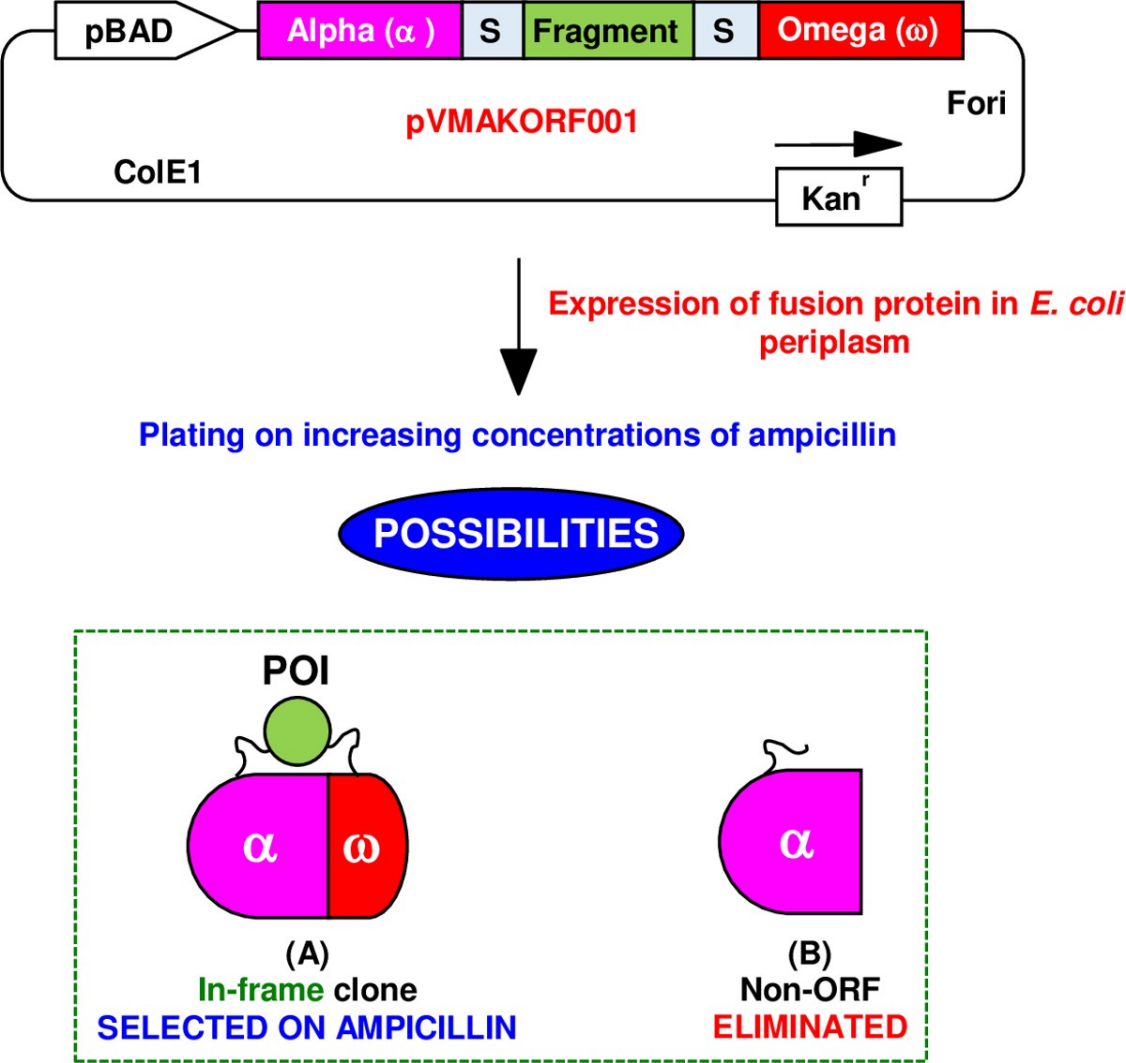
Concept for ORF selection using split beta-lactamase protein fragment complementation-based vector system pVMAKORF001 in *E*. *coli*. Depending on the frame and solubility of the fragment cloned between two parts of beta-lactamase, it can have two fates. (A) If the cloned fragment encodes an in-frame protein, it will reconstitute beta-lactamase activity and will grow on ampicillin containing media plate depending on its solubility. (B) If the cloned fragment encodes an off-frame protein, it will not produce full-length beta-lactamase and the clone will be eliminated upon selection on ampicillin containing media plate.

The aim of this study was to develop a robust system for the ORF selection of DNA fragment libraries. The system described here is based on the periplasmic *E*. *coli* enzyme TEM-1 beta-lactamase, which is responsible for conferring the resistance against beta-lactam drugs like ampicillin. This enzyme has been dissected into two protein fragments, namely Bla-Alpha (24–195 amino acids) and Bla-Omega (196–286 amino acids) [29, 32]. These fragments are known to not interact by themselves in isolation but can re-constitute the enzymatic activity when brought together by a mediator. This mediator can be an interacting protein pair attached to the two beta-lactamase fragments or a sequence encoding a well-folded soluble protein inserted in-frame between the two fragments. The ORF selection system described here is based on the premise that when the gene of interest (GOI) is inserted between the two fragments of TEM-1 beta-lactamase gene, it can have two possible fates (Fig 1). If the GOI is in-frame with Bla-Alpha and Bla-Omega, it would allow the translation of the complete beta-lactamase molecule and the two fragments of beta-lactamase will interact to confer resistance against ampicillin (Fig 1A). On the other hand, if the GOI carries stop-codons, this would lead to termination of the translation, and the resulting incomplete beta-lactamase fusion protein would not confer any resistance against ampicillin (Fig 1B). Thus, in principle, plating of clones on appropriate concentration of ampicillin allows selection of clones encoding in-frame sequences.

### Development and characterization of the ORF selection system

**Fig 2:**
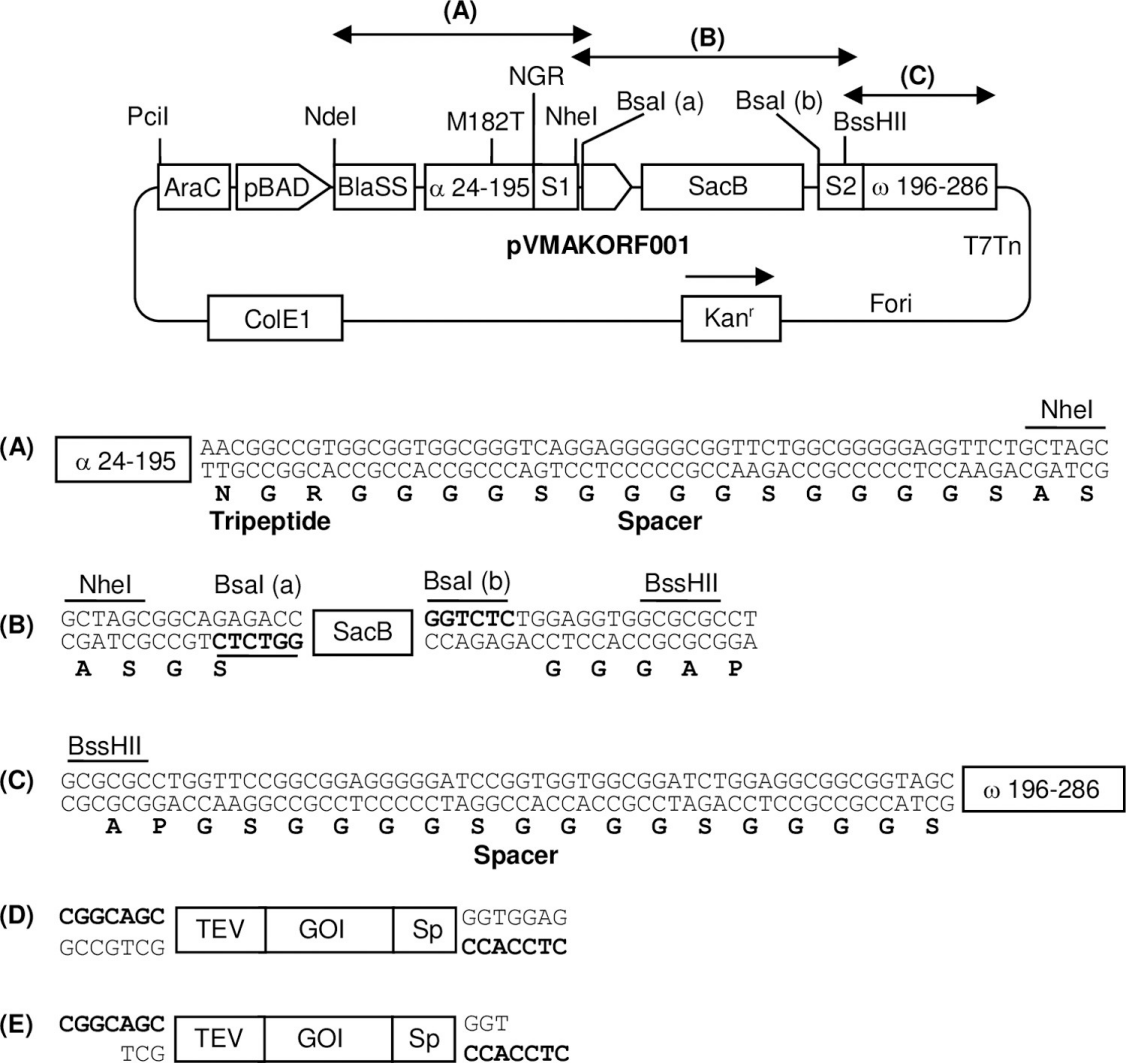
Schematic representation of pVMAKORF001 vector. Only relevant genes and restriction sites are shown. The map is not to scale. Kan^r^, kanamycin resistance gene; ColE1, origin of replication; Fori, phage M13 origin of replication; AraC and pBAD, Cassette encoding arabinose promoter and its regulator AraC; BlaSS, Native bla signal sequence; α 24–195, Bla-Alpha fragment encoding 24–195 amino acids of beta-lactamase; M182T, Methionine 182 to threonine mutation; NGR, Asparagine-Glycine-Arginine tripeptide; S1 and S2, (Gly4Ser)3 linker; Sp. Spacer sequence; TEV, Tobacco Etch Virus protease sequence; ω 196–286, Bla-Omega fragment encoding 196–286 amino acids of beta-lactamase; SacB and SacR, gene cassette encoding levansucrase of *B*. *subtilis*. (A)-(C) Sequence of different cassettes of the vector. (D) Sequence of insert to be cloned in the vector. (E) Insert carrying 4 base 5’ overhangs after T4 DNA polymerase treatment in the presence of dTTP.

To test the ORF selection system, a vector pVMAKORF001 was constructed. This is a medium copy number vector compatible with restriction enzyme-free cloning and contains a 2.0 kb counter-selection marker SacB as stuffer flanked by two appropriately oriented BsaI restriction enzyme sites (Fig 2). The inserts carrying vector compatible 5’-4 base overhangs can be cloned in the place of SacB stuffer after the digestion of vector with BsaI enzyme, which would generate 5’-4 base non-cohesive overhangs to allow directional cloning of appropriately prepared DNA fragments (Fig 2) [35]. This vector allows arabinose-regulated expression of split-beta-lactamase fusion proteins (Alpha-POI-Omega) and secretion into the *E*. *coli* periplasm.

For the characterization of this system, two in-frame clones encoding a spacer (Alpha-Spacer-Omega, positive control) or *M*. *tuberculosis* 19 kDa protein (Alpha-19kDa-Omega, positive control) between Bla-Alpha and Bla-Omega, and an off-frame clone encoding a stop codon (Alpha-Stop, negative control) between Bla-Alpha and Bla-Omega were constructed (S1A Fig). Western blot analysis of periplasmic fraction of both positive controls showed good expression, whereas, no band was detected in negative control (Alpha-Stop) (S1C Fig). This suggests that in-frame constructs successfully translocate to periplasm followed by reconstitution of beta-lactamase activity, thus conferring ampicillin resistance.

**Fig 3:**
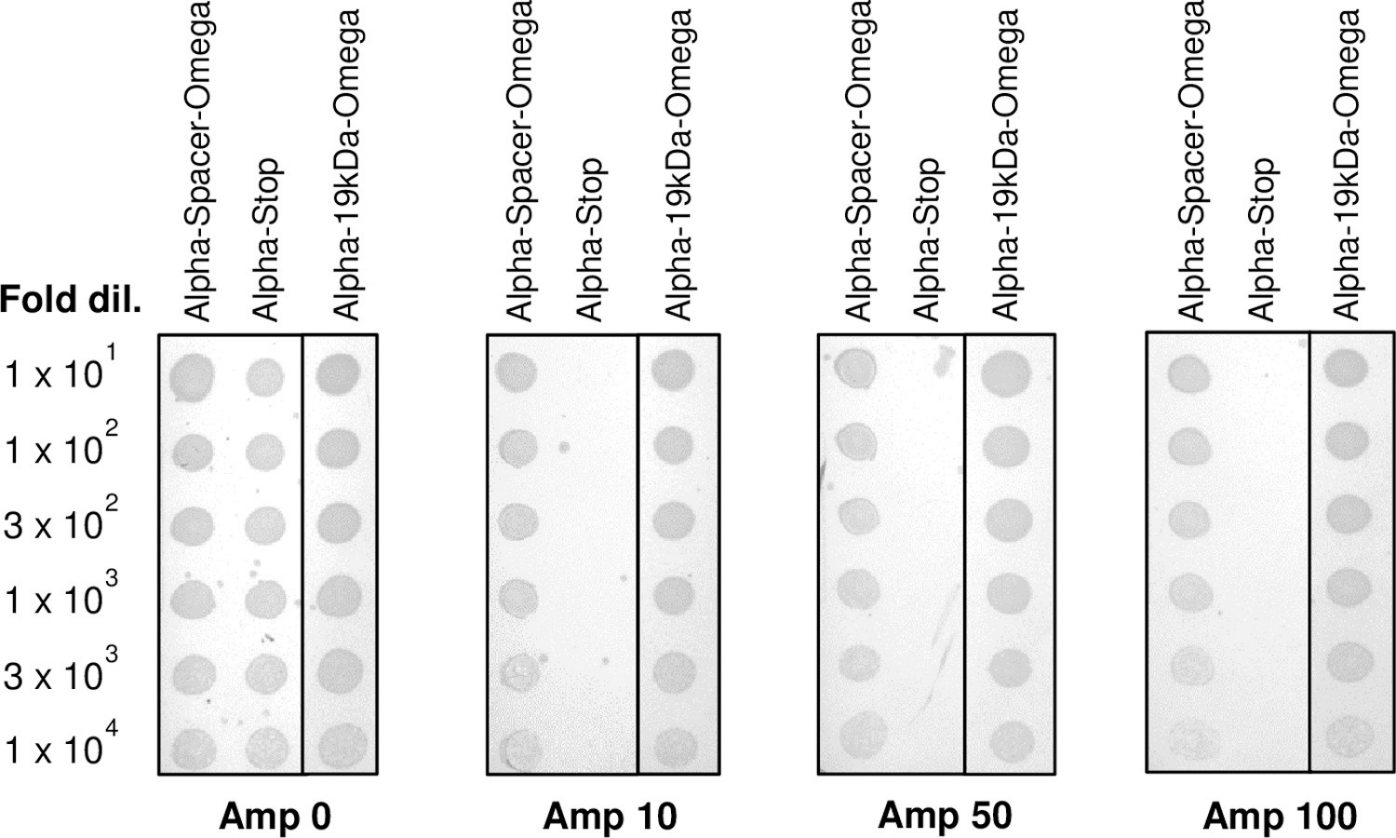
ORF selection profile of model constructs upon selection on increasing concentrations of ampicillin. Clones encoding in-frame Alpha-Spacer-Omega, Alpha-19kDa-Omega, and off-frame Alpha-stop proteins were pre-induced with 0.0002% arabinose, and serial dilutions were spotted on plates containing LB media with 0.0002% arabinose and increasing concentrations of ampicillin (0 μg/ml to 100 μg/ml).

To determine a concentration of ampicillin suitable for ORF selection of the in-frame clones, the three clones (Alpha-19kDa-Omega, Alpha-Spacer-Omega, Alpha-Stop) were grown at 37°C on plates containing increasing concentrations of ampicillin. The in-frame clones encoding a spacer or 19 kDa protein between Bla-alpha and Bla-Omega were found to survive even up to 100 μg/ml ampicillin, whereas, the off-frame clone was eliminated at 10 μg/ml ampicillin (Fig 3). Based on this, it was concluded that the off-frame clones could be eliminated using 10 μg/ml ampicillin.

### Optimization of ORF selection with respect to DNA fragment libraries

During the construction of DNA fragment libraries, statistically only one out of 18 clones is in correct reading frame, and therefore most of the clones are off-frame. Thus, the conditions for ORF selection were optimized to mimic a situation experienced with the DNA fragment libraries. The in-frame clone (Alpha-19kDa-Omega) and the off-frame clone (Alpha-Stop) were grown and mixed in 1: 20 ratio followed by selection at 37°C in the absence/presence of 10 μg/ml ampicillin. Colony PCR-based analysis of 24 clones obtained on non-ampicillin plate revealed that only 1 clone carried in-frame 19 kDa and rest, 23 were off-frame clones (since initially mixed in ~1:20) (Table 1 and S2 Fig). Whereas, the analysis of 72 clones selected at 10 μg/ml ampicillin revealed that 71/72 clones carried the in-frame 19 kDa (1 clone did not show amplification) (Table 1 and S2 Fig). The result clearly showed that the split beta-lactamase protein fragment complementation-based ORF selection system is highly robust and efficient for the selection of in-frame clones and can be successfully applied for ORF selection of DNA fragment libraries.

**Table 1:** Efficiency of ORF selection with a culture mix mimicking ORF to non-ORF ratios observed with DNA fragment libraries.

	Alpha-Stop (Off-frame, 197 bp)	Alpha-19 kDa-Omega (In-frame, 689 bp)
Amp 0 μg/ml	23/24	1/24
Amp 10 μg/ml	0/72	71/72

Cultures of clones encoding in-frame Alpha-19 kDa-Omega and off-frame Alpha-stop proteins were mixed in 1:20 ratio, respectively and plated on LB media supplemented with 0.0002% arabinose and two concentrations of ampicillin (0 and 10 μg/ml) and incubated at 37°C for 16 hr. The table shows the summary of distribution of clones.

### Construction and characterization of *M*. *tuberculosis* gene fragment library-MTBLIB42

The ORF selection system was finally validated by ORF selection of a *M*. *tuberculosis* 30 gene fragment library. Since one of the focus of our laboratory is in developing antibody-based diagnostics for tuberculosis detection, *M*. *tuberculosis* genes reported to have significant immuno-diagnostic importance were identified from the literature. A gene fragment library of 30 *M*. *tuberculosis* genes fulfilling this criterion was constructed in the ORF selection vector pVMAKORF001. The final aim was to produce an ORF-selected *M*. *tuberculosis* 30 gene fragment library encoding gene fragments in the size range of ~150–400 bp (approximately 50–133 amino acids) that can provide ready access to fragments spanning across the 30 selected proteins encoded by *M*. *tuberculosis* genome for different applications including identification of immunodominant regions using TB patient sera [2, 6, 15] or epitope mapping of antibodies [10]. The library was constructed using a previously described protocol with appropriate modifications including the use of emulsion PCR and improved DNA manipulation techniques to obtain superior quality libraries (S3 Fig) [35]. For the construction of library, 30 genes were amplified without stop codon and subjected to shearing (S4 and S5 Figs). The fragments were processed as depicted in S3 Fig and cloned in the ORF selection vector to obtain a library comprising of 8 x 10^7^ clones. This unselected library of primary transformants was named as MTBLIB42C01. PCR-based analysis of 24 randomly selected clones revealed that 100% clones were recombinants and in the desired size range of ~150–400 bp. DNA sequence analysis of 23 clones further revealed that clones spanned uniformly across 30 genes and large number of clones (16/23; 69.5%) were off-frame.

### ORF selection of MTBLIB42C01

Based on the optimizations (Table 1 and Fig 3), 10 μg/ml ampicillin allowed the selection of clones encoding in-frame DNA fragments. For improved selection efficiency, we used 20 μg/ml ampicillin for ORF selection. Analytical scale ORF selection revealed approximately 50-fold decrease in the number of colonies upon selection. PCR-based analysis of 32 clones obtained on 20 μg/ml ampicillin revealed that 100% clones were recombinants and DNA sequence analysis revealed all the clones were in-frame and spanned uniformly across the 30 *M*. *tuberculosis* genes. Based on these results, preparative-scale ORF selection of MTBLIB42C01 was performed at 20 μg/ml ampicillin to obtain an ORF-selected library of ~4 x 10^5^ clones, named as MTBLIB42C02.

### Characterization of MTBLIB42C01 and MTBLIB42C02 libraries using next-generation sequencing

**Fig 4:**
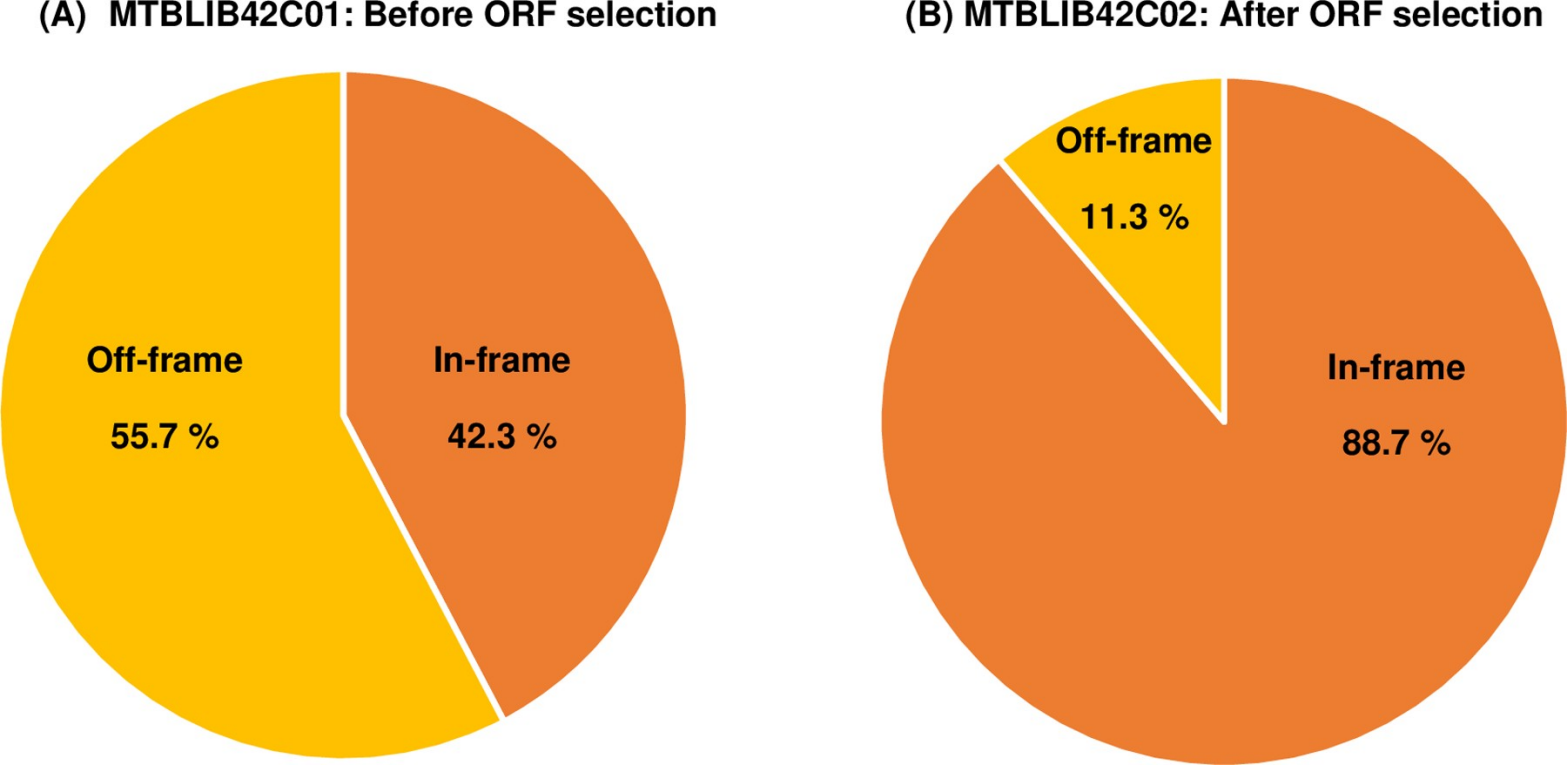
Analysis of ORF selection efficiency by high-throughput sequencing of MTBLIB42 before and after ORF selection. MTBLIB42C01 and MTBLIB42C02 libraries were sequenced using MiSeq Nano v2 chemistry for 2 x 250 cycles. A total of 5,66,496 (MTBLIB42C01) and 5,38,956 (MTBLIB42C02) merged reads aligned to 30 *M*. *tuberculosis* genes, and the data was further analyzed using in-house developed pipeline, ORFSELECT. (A) MTBLIB42C01, (B) MTBLIB42C02. However, the number of in-frame clones in the unselected library was higher (42.3%) as compared to the theoretically possible number (5.6%; 1 in 18 is in-frame). To further investigate this difference, we generated overlapping 30 gene fragment libraries of 100 bp, 200 bp and 300 bp fragment size with 1 bp incremental shift *in silico* and determined the number of theoretically possible in-frame clones. The number of in-frame clones for theoretical 30 gene fragment libraries of size 100 bp, 200 bp and 300 bp was found to 58.3%, 40.9%, and 31.9%, respectively, which corroborated with our experimental findings.

To further assess the efficiency of ORF selection at genome-scale, both the libraries (before and after selection) were subjected to high-throughput sequencing (S6 Fig). About 0.5 million reads were analyzed and 42.3% reads were found to encode in-frame sequences in the unselected MTBLIB42C01, whereas 88.7% reads were found to encode in-frame sequences post ORF selection of MTBLIB42C02 library indicating towards successful ORF selection at library scale (Fig 4).

### Expression analysis of ORF-selected fragments in MTBLIB42C02

**Fig 5:**
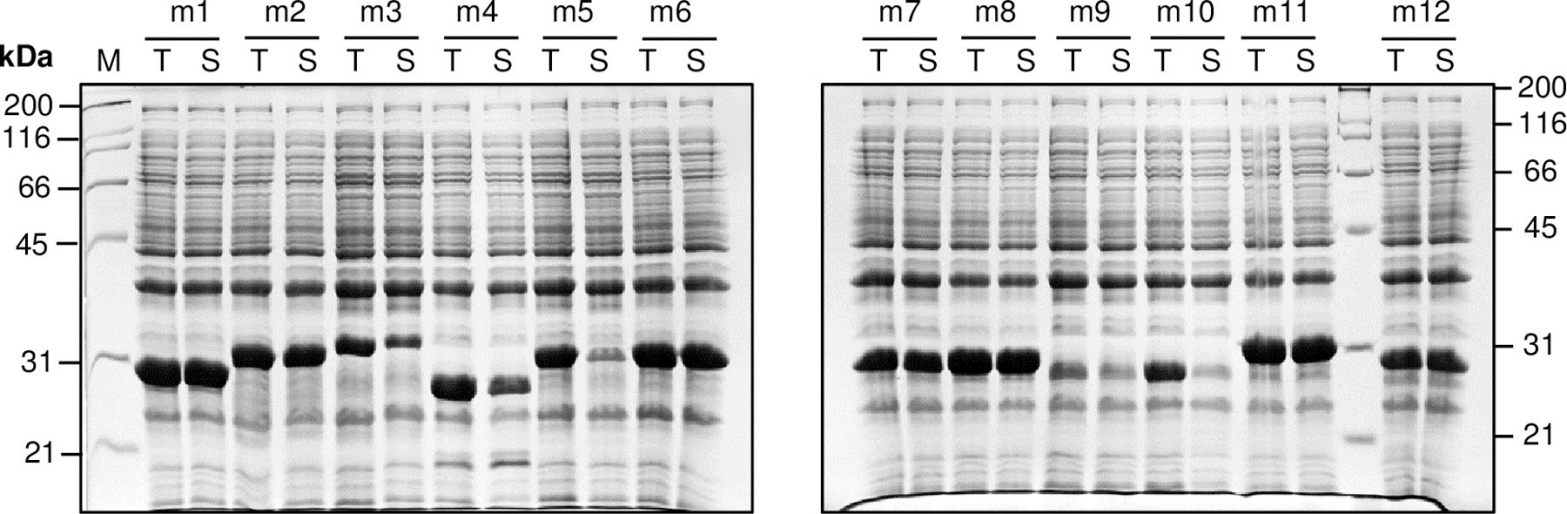
Expression and solubility analysis of 12 randomly selected clones obtained after transfer of MTBLIB42C02 in pVMH10D-BAP001 expression vector. Twelve randomly selected clones were subjected to auto-induction at 18°C and solubility was assayed using PopCulture assay. The total cell (T) and soluble (S) fractions were analyzed using SDS-PAGE.

To assess the potential of ORF-selected MTBLIB42C02 library as a source of protein fragments representing 30 mycobacterial genes, we checked the expression and solubility profile of ORF-selected fragments. For this, the ORF-selected fragments were amplified using adapter-specific primers (L3-s and K2-s) flanking the fragments and cloned into an expression vector pVMH10D-BAP001 using restriction-enzyme free cloning strategy to obtain MTBLIB42C02 expression library (S7 Fig). PCR analysis of 16 randomly selected clones from the expression library showed that 100% clones were recombinants and sequence analysis of 15 clones revealed that 14/15 clones were in correct reading frame. Out of these, 12 clones were subjected to expression analysis and 11/12 clones exhibited good expression (Fig 5). One clone (m9) showed low expression. Furthermore, 10/12 clones expressed highly soluble protein in cytosol and 2/12 clones (m5 and m10) showed relatively low solubility. Overall, the results clearly indicated that split beta-lactamase system is efficient in ORF selection and ORF-selected clones show good soluble expression with appropriate expression vector. Such protein fragments can be valuable in different applications including antibody characterization and epitope mapping.

## Discussion

This paper describes the development and optimization of a robust ORF selection system for DNA fragment libraries, which is based on split beta-lactamase based protein fragment complementation. The system has been successfully employed for the generation of an ORF-selected *M*. *tuberculosis* gene fragment library. This system is based on a previously described system for the selection of stabilized variants of Immunity protein 7, and identification of chemicals that enhance the solubility of proteins [31, 47, 48]. However, the application of this system for ORF selection of large DNA fragment libraries is novel to the best of our knowledge. In addition, the process of DNA fragment preparation and library construction is very robust. Further, the paper also provides a method for transferring the ORF-selected DNA fragments into an expression vector using a well-optimized restriction enzyme-free cloning strategy, for soluble cytosolic expression. The ORF selection system reported here employs a medium copy number pVMAKORF001 vector, which allows the periplasmic expression of split beta-lactamase fusion proteins under the control of tightly regulated arabinose-inducible pBAD promoter.

Several groups have also described ORF selection systems that employ TEM-1 beta-lactamase, albeit in a different configuration, where the POI is inserted between signal sequence and full-length beta-lactamase enzyme and the level of resistance conferred against ampicillin is directly proportional to the solubility of the POI [1, 20, 26–28]. However, since beta-lactamase *per se* is highly soluble, the degradation of POI in the periplasm can lead to the release of full-length beta-lactamase that can confer resistance equal to wild-type enzyme, contributing to false-positives. Since our system is based on two fragments of the beta-lactamase, namely Bla-Alpha and Bla-Omega, in cases where the POI is degraded, these two fragments will be inactive by themselves, making it highly robust and compatible with both ORF selection as well as faithful solubility selection on increasing concentrations of ampicillin (not shown in this study) with significantly less false-positives as exemplified earlier [31].

Several phage-based systems have also been described in the literature for ORF selection. Gupta et al., have described the use of Fd-tet phage-based system for the construction of ORF-selected gene fragment libraries, where only the phages encoding DNA fragment in-frame with g3 were infectious and could be selected by infection in *E*.*coli* [21]. Similarly, a phage-packaging system comprising of a g3-deficient Hyperphage has been successfully employed for ORF selection, in which the only source of g3p is the fusion protein encoded by the phagemid DNA, thus only the clones carrying in-frame fusion-g3p protein can produce infectious phage particles [22, 23]. However, apart from the low phage titers produced by Hyperphage as compared to VCSM13 helper phage, it has been found to cause a reduction in the average size distribution of the ORF-selected library from original 50–700 bp to 50–300 bp [14]. Gupta et al., have also described a novel helper phage-AGM13, which carries trypsin-sensitive sites in g3, due to which the phages encoding in-frame ORFs can be selected by trypsin-treatment of the rescued phage population, followed by infection in *E*. *coli* [24]. However, the simple one-step ORF selection system described in the current work is expected to be superior to the other systems including helper phage-based ORF selection systems that require elaborate protocols of phage-rescue, purification, re-infection, etc. to obtain ORF-selected libraries [10, 21, 23].

The ORF selection system described here allows selection of in-frame clones at ampicillin concentrations of 10 μg/ml or above. It is efficient even when the ratio of in-frame: off-frame clones is 1 in 20, as observed during the construction of DNA fragments libraries. The utility of the system has been demonstrated by construction of an ORF-selected gene fragment library of 30 mycobacterial genes of immunodiagnostic importance. The *M*. *tuberculosis* 30 gene fragment library, MTBLIB42C01 (200–400 bp) was constructed in the ORF selection vector using a modification of the highly robust protocol described before to obtain superior quality library [35]. The unselected library was found to contain 30% and 42.3% in-frame clones using Sanger’s sequencing and NGS, respectively. It was noted that in the unselected library, MTBLIB42C02, the percentage of in-frame clones was higher than theoretically calculated value (~ 5%, 1/18). To understand this variation, the number of overlapping in-frame clones in the 30 gene fragment library was calculated *in silico*, which lies between 30–60% for a library of 100–300 bp. This suggests that the high number of in-frame clones in the unselected library is because of high GC content of the source DNA (~ 65%) and small size of fragments, due to which likelihood of hitting stop codon is minimal.

Upon ORF selection, the library was found to contain 100% and 88.7% in-frame clones using Sanger’s sequencing and NGS, respectively. The careful analysis of sequences obtained with Sanger’s sequencing revealed that in about 10% clones, the 5’ TEV adapter sequence carried 1–2 bp deletions, which led to correction of the frame of otherwise off-frame gene-fragments and consequent selection of such clones at 20 μg/ml ampicillin. We believe that during re-amplification of fragments for NGS, the adapter sequence of such clones gets corrected by the NGS primer and they get identified as off-frame fragments during data analysis contributing to slight discrepancy in the data obtained with Sanger’s sequencing and NGS.

The cloning strategy described in this study provides a universal and smooth restriction enzyme-free transfer of selected fragments to any other vector of interest at genome-scale [35]. In this study, we have successfully demonstrated the transfer of ORF-selected fragments into an expression vector for the production of soluble protein fragments. Since the system is based on the selection in *E*. *coli* periplasm, it is particularly useful for the ORF selection of large DNA fragment libraries, as it can also allow the selection of proteins carrying disulphide bonds as compared to other systems based on selections in *E*. *coli* cytosol [19].

The ORF selection system described here is expected to find use in numerous applications including the construction of high-quality ORF-selected DNA fragment libraries, which can be employed for the functional annotation of the large number of genes identified during high-throughput genome sequencing projects over last two decades [1]. Alternatively, such ORF-selected libraries can also be transferred into appropriate expression vectors to express protein fragments as a mixture, which can be used for several applications including the identification of antibody epitopes by incubation of the mixture with antibodies, followed by immunoprecipitation and identification of target proteins using MS. Such libraries can also be transferred to different surface display systems for identification of immunodominant epitopes using patient sera [15–18, 49]. Zantow et al., have employed ORF-selected DNA fragment libraries of gut microbiome to study protein-protein interactions and identify novel biomarkers [12]. This new ORF selection system will also find use in the enrichment of functional clones during construction of large phage-displayed human antibody libraries to improve the efficiency of specific antibody binders as described previously using full-length beta-lactamase [27, 50, 51].

In summary, this paper describes a robust split beta-lactamase-based system for ORF-selection at genome-scale with a strategy to transfer ORF-selected DNA fragments to other compatible vectors.

## Supporting information

S1 Fig Analysis of the expression of beta-lactamase fusion proteins in *E*. *coli* TOP10F’ periplasm. Clones encoding in-frame Alpha-Spacer-Omega, Alpha-19kDa-Omega and off-frame Alpha-Stop proteins were induced with 0.05% arabinose and periplasmic fraction was isolated. Dilutions of periplasm were analysed (Neat, 1:2, and 1:4) by Western blot with anti-Omega MAb BA09-3. (A) Details of the proteins analyzed. (B) Site where MAb BA09-3 binds. (C) Western blot analysis.
(PDF)

S2 Fig Analysis of the efficiency of the ORF selection with a culture mix mimicking ORF to non-ORF ratios observed with genome fragment libraries. Cultures of clones encoding in-frame Alpha-19kDa-Omega and off-frame Alpha-stop proteins were mixed in 1:20 ratio, and plated on LB media supplemented with 0.0002% arabinose and two concentrations of ampicillin (0 and 10 μg/ml) and incubated at 37°C for 16 hours. (A)-(C). 24 and 72 colonies obtained on plates carrying 0 μg /ml and 10 μg/ml ampicillin concentrations, respectively were analyzed using colony PCR and products were analyzed on 1.2% agarose gel (M, 1 Kb plus DNA ladder, Invitrogen; Lane 1–24, PCR amplicons of 24 colonies picked from plates carrying 0 μg/ml ampicillin; Lane 25–96, PCR amplicons of 72 colonies picked from plates carrying 10 μg/ml ampicillin).
(PDF)

S3 Fig Strategy for the construction of *M*. *tuberculosis* DNA fragment library. (A) Preparation of inserts for library. (A) *M*. *tuberculosis* genomic DNA or PCR amplified DNA (individual coding sequences) are sheared using acoustics-based ultrasonicator followed by desired size-selection using agarose gel (Step A). The size-selected DNA fragments are subjected to end-repair with T4 DNA polymerase and 5’ phosphorylation with T4 polynucleotide kinase followed by 3’ A-tailing using Klenow fragment (exo-) (Step B). The A-tailed DNA is ligated to desired adapters carrying 3’ T-tails (Step C). After ligation, the adapter ligated DNA is subjected to nick-repair using Bst polymerase (Step D) followed by selection using streptavidin-coated magnetic beads to eliminate fragments carrying same adapter on either end (Step E-F). The ssDNA fragments carrying different adapters on either end are then used as a template for emulsion PCR to obtain dsDNA pool of inserts (Step G). (B) Cloning of inserts in ORF selection vector. The inserts obtained in (A) are subjected to treatment with T4 DNA polymerase in the presence of dTTP to generate non-compatible and non-palindromic 4 base 5’ overhangs on either ends of the inserts. The inserts are then ligated to BsaI-digested pVMAKORF001 vector (for ORF selection) using T4 DNA ligase. The ligation mix is then electroporated in *E*. *coli* host TOP10F’ to obtain *M*. *tuberculosis* DNA fragment library.
(PDF)

S4 Fig Analysis of 30 *M*. *tuberculosis* genes amplified using PCR. The 30 genes were divided into 3 groups and after PCR amplification and QIAquick PCR/gel-based purification, an aliquot was analysed on agarose gel. (A) Group A genes. (B) Group B genes. + SS, with signal sequence. (C) Group C genes. Also see S1 Table for details of genes.
(PDF)

S5 Fig Construction of 30 *M*. *tuberculosis* gene fragment library MTBLIB42C01. (A) Analysis of preparative sheared pool of 28 *M*. *tuberculosis* genes using agarose gel. The 28 genes (excluding CFP10 and ESAT-6) were pooled and sheared using Covaris ultrasonicator to obtain 100–500 bp fragments as per manufacturer’s instructions. Lane M, 50 bp DNA ladder; Lane 1, Sheared DNA pool. (B) Agarose gel-based size selection of the sheared DNA. Size selection of the sheared DNA was performed using 1.2% SYBR safe agarose gel to obtain fragment in the size range of 150–400 bp. Lane M, 50 bp DNA ladder; Lane 1, Size selected DNA. (C) Analysis of DNA fragments before and after adapter ligation. Lane M, 1 kb DNA ladder; Lane 1, before adapter ligation; Lane 2, after adapter ligation. (D) Analysis of T4 DNA polymerase-treated insert. Lane M, 50 bp DNA ladder; Lane 1, T4 DNA polymerase-treated 220–380 bp insert. (E) Analysis of BsaI-digested vector pVMAKORF001. M-1 kb DNA ladder, Lane 1, 2 and 3- Dilutions of the digested vector pVMAKORF001. (F) Workflow for the construction and storage of MTBLIB42C01 library.
(PDF)

S6 Fig Strategy and primer design for preparation of dual-indexed MTBLIB42C01 and MTBLIB42C02 gene fragment libraries for NGS using Illumina MiSeq platform. (A) PCR strategy based on 2 overlapping primers based on primers described by Illumina but with modified outer indexed primers for improved annealing to the inner primer. (B) Details of the primers employed during amplification. * indicates phosphorothioate bond. (C) Analysis of MTBLIB42C01 library amplified using emulsion PCR. Lane M, 1 kb DNA ladder; Lane 1, MTBLIB42C01 dual-indexed library. (D) Analysis of MTBLIB42C02 library amplified using emulsion PCR. Lane M, 1 kb DNA ladder; Lane 1, MTBLIB42C02 dual-indexed library
(PDF)

S7 Fig Schematic representation of strategy for transfer of ORF selected gene fragments from MTBLIB42C02 into pVMH10D-BAP001 expression vector. Only relevant genes and restriction sites are shown. The maps are not to scale. Amp^r^, ampicillin resistance marker; ColE1, origin of replication; Fori, phage M13 origin of replication. RBS, Ribosome binding site; T7PO, T7 promoter-operator; H10, Deca-histidine tag; D, Bacteriophage Lambda coat protein D; TEV, Tobacco etch virus protease cleavage site; SacB, 2 kb *B*. *subtilis* levansucrase gene; BAP, Biotin Acceptor Peptide tag; S, spacer; GOI, Gene of interest. Sp, Glycine-serine rich spacer. (A) Gene fragments (GOI) appended by DNA sequence encoding TEV protease site (TEV) and spacer sequence (Sp) at 5’ and 3’ ends, respectively, were amplified using emulsion PCR from MTBLIB42C02, treated with T4 DNA polymerase in the presence of dTTP, and cloned into BsaI-digested pVMH10D-BAP001 vector. (B)-(C) Sequence of different components of pVMH10D-BAP001 expression vector (as denoted in the map).
(PDF)

S1 Table Details of PCR based amplification of 30 *M*. *tuberculosis* genes and their pooling for shearing to obtain DNA fragments. * denotes that these genes were purified from agarose gel, rest were purified using QIAquick PCR purification kit. NA denotes that these genes were not pooled in the gene mix subjected to shearing. # denotes that these genes were added as full-length genes in 10 μg 200–400 bp sheared DNA.
(PDF)

S2 Table Details of the primers employed for the amplification of 30 *M*. *tuberculosis* genes. The primers were designed for amplification of 30 genes without stop codon using *M*. *tuberculosis* genomic DNA as the template. Rv0934 (38 kDa) gene was amplified using 2 different 5’ primers with same 3’ primer to obtain gene with and without signal sequence. Rv1837c (81 kDa) was amplified using two step PCR.
(PDF)

S3 Table Details of the primers used for the preparation of adapter duplex and amplification of single or double stranded DNA fragment library. Bases in blue indicate the additional 7-bases required to generate 4 base 5’ BsaI compatible overhangs after T4 DNA Polymerase treatment in presence of dTTP. Bio denotes Biotin moiety attached to the 5’ end of the primer Bio L1-s. * denotes one phosphorothioate bond present at the 3’-end of both the amplification primers. The amino acid sequence encoded by the template to which primers anneal is shown in bold.
(PDF)

S1 Raw images (PDF)
