# Supplementary material for: An efficient ORF selection system for DNA fragment libraries based on split beta-lactamase complementation
Source: PLoS One. 2020 Jul 23;15(7):e0235853. doi: 10.1371/journal.pone.0235853 (PMC7377443; doi:10.1371/journal.pone.0235853)

(A)

S. No.	Construct	Protein expressed	Size (kDa)	Solubility
1	pVMAKORFWOI-In001	Alpha-Spacer-Omega	31.6	~ 100 %
2	pVMAKORF19kDa001	Alpha-19kDa-Omega	47.1	~ 100 %
3	pVMAKORFWOI-Off001	Alpha-stop	NA	NA

(B)

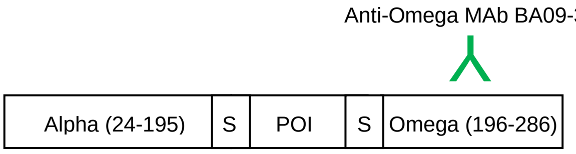

(C)

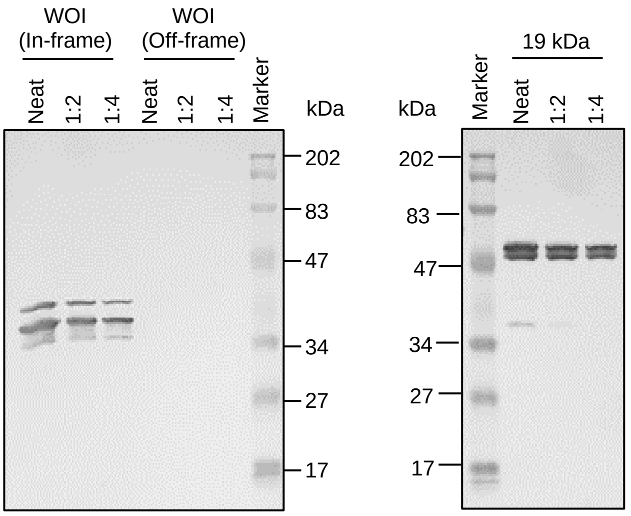

Supplement: S1 Fig — Clones encoding in-frame Alpha-Spacer-Omega, Alpha-19kDa-Omega and off-frame Alpha-Stop proteins were induced with 0.05% arabinose and periplasmic fraction was isolated. Dilutions of periplasm were analysed (Neat, 1:2, and 1:4) by Western blot with anti-Omega MAb BA09-3. (A) Details of the proteins analyzed. (B) Site where MAb BA09-3 binds. (C) Western blot analysis. (PDF) [file pone.0235853.s001.pdf]
