# Supplementary figures and images for: An efficient ORF selection system for DNA fragment libraries based on split beta-lactamase complementation
Source: PLoS One. 2020 Jul 23;15(7):e0235853. doi: 10.1371/journal.pone.0235853 (PMC7377443; doi:10.1371/journal.pone.0235853)

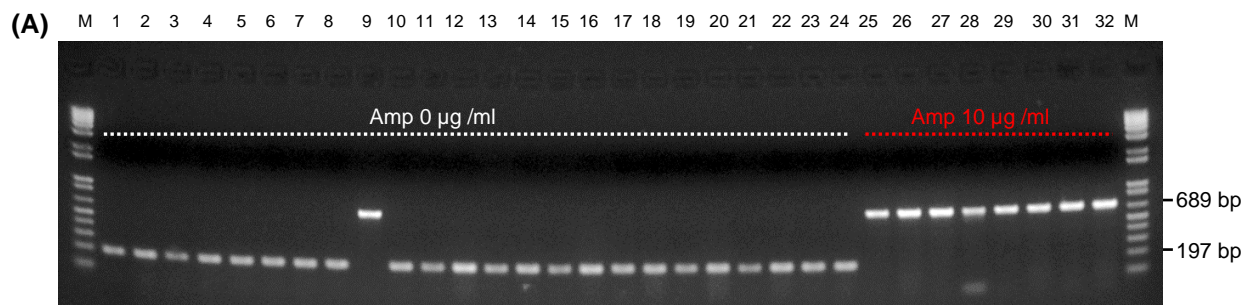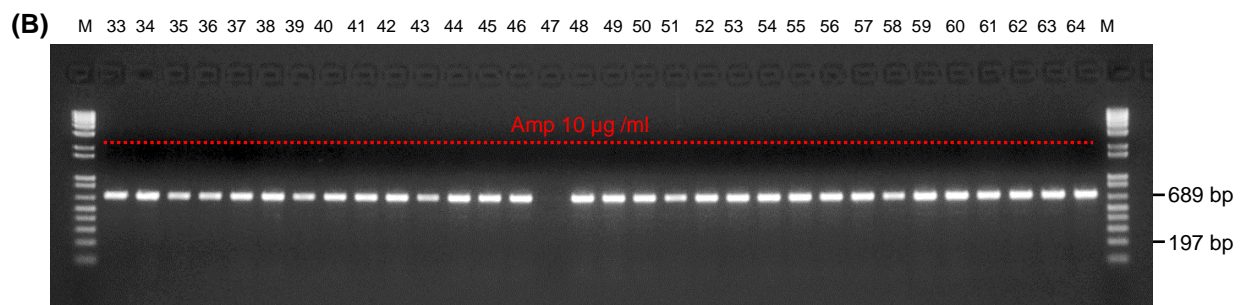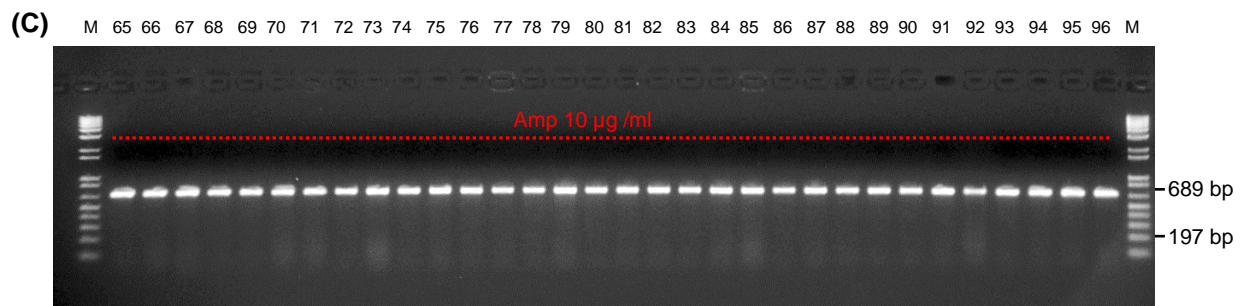

Supplement: S2 Fig — Cultures of clones encoding in-frame Alpha-19kDa-Omega and off-frame Alpha-stop proteins were mixed in 1:20 ratio, and plated on LB media supplemented with 0.0002% arabinose and two concentrations of ampicillin (0 and 10 μg/ml) and incubated at 37°C for 16 hours. (A)-(C). 24 and 72 colonies obtained on plates carrying 0 μg /ml and 10 μg/ml ampicillin concentrations, respectively were analyzed using colony PCR and products were analyzed on 1.2% agarose gel (M, 1 Kb plus DNA ladder, Invitrogen; Lane 1–24, PCR amplicons of 24 colonies picked from plates carrying 0 μg/ml ampicillin; Lane 25–96, PCR amplicons of 72 colonies picked from plates carrying 10 μg/ml ampicillin). (PDF) [file pone.0235853.s002.pdf]

(A)

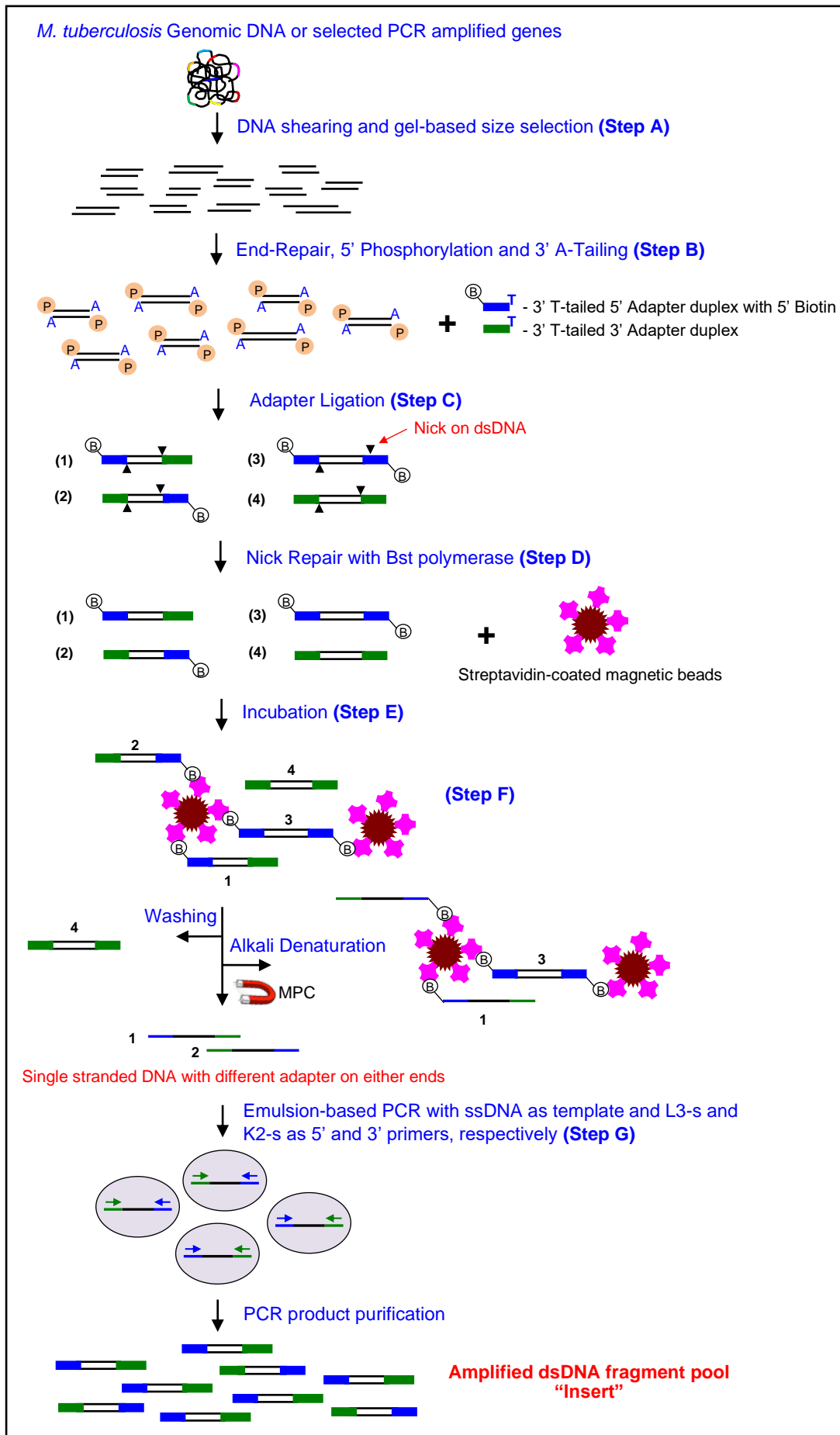

(B)

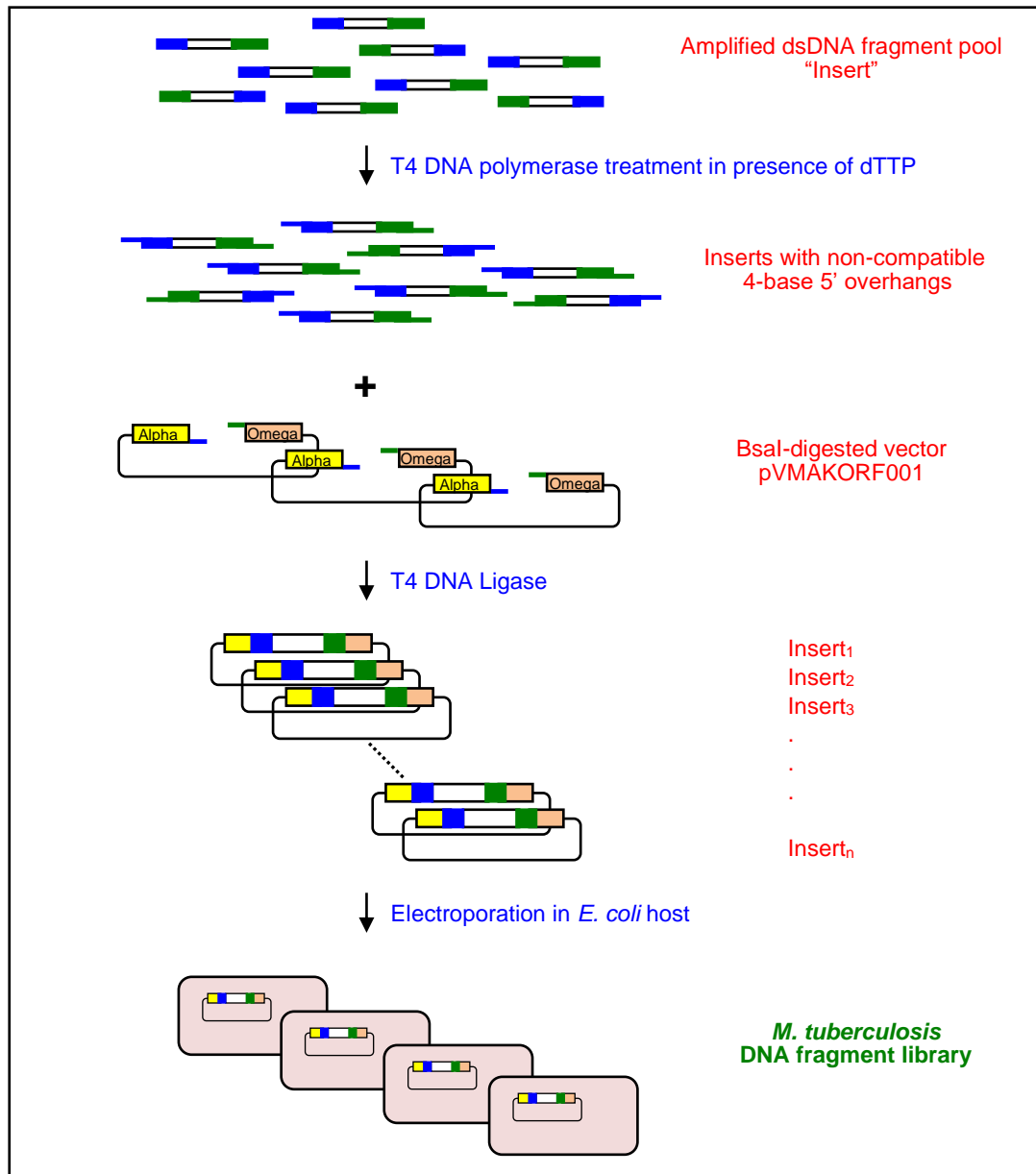

Supplement: S3 Fig — (A) Preparation of inserts for library. (A) M. tuberculosis genomic DNA or PCR amplified DNA (individual coding sequences) are sheared using acoustics-based ultrasonicator followed by desired size-selection using agarose gel (Step A). The size-selected DNA fragments are subjected to end-repair with T4 DNA polymerase and 5’ phosphorylation with T4 polynucleotide kinase followed by 3’ A-tailing using Klenow fragment (exo-) (Step B). The A-tailed DNA is ligated to desired adapters carrying 3’ T-tails (Step C). After ligation, the adapter ligated DNA is subjected to nick-repair using Bst polymerase (Step D) followed by selection using streptavidin-coated magnetic beads to eliminate fragments carrying same adapter on either end (Step E-F). The ssDNA fragments carrying different adapters on either end are then used as a template for emulsion PCR to obtain dsDNA pool of inserts (Step G). (B) Cloning of inserts in ORF selection vector. The inserts obtained in (A) are subjected to treatment with T4 DNA polymerase in the presence of dTTP to generate non-compatible and non-palindromic 4 base 5’ overhangs on either ends of the inserts. The inserts are then ligated to BsaI-digested pVMAKORF001 vector (for ORF selection) using T4 DNA ligase. The ligation mix is then electroporated in E. coli host TOP10F’ to obtain M. tuberculosis DNA fragment library. (PDF) [file pone.0235853.s003.pdf]

(A)

**Group A; 17 Genes (285-800 bp)**

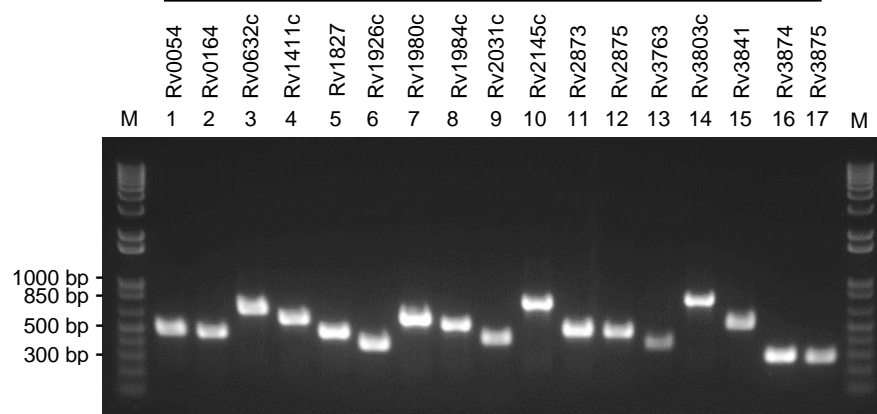

(B)

**Group B; 11 Genes (801-1400 bp)**

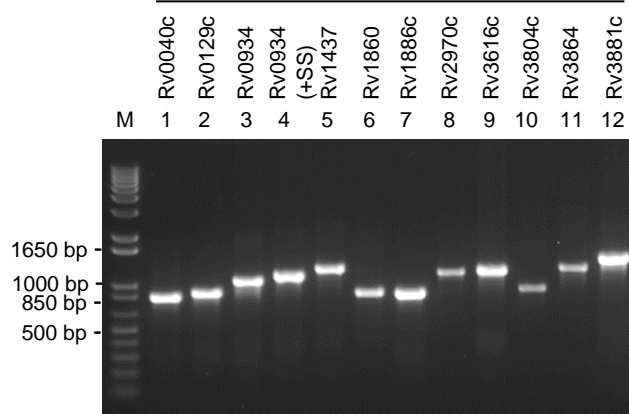

**(C) Group C; 2 Genes (1401-2500 bp)**

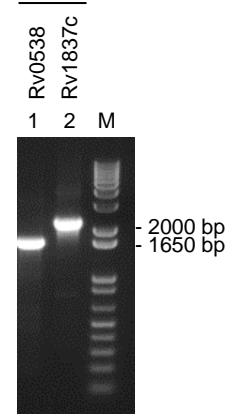

Supplement: S4 Fig — The 30 genes were divided into 3 groups and after PCR amplification and QIAquick PCR/gel-based purification, an aliquot was analysed on agarose gel. (A) Group A genes. (B) Group B genes. + SS, with signal sequence. (C) Group C genes. Also see S1 Table for details of genes. (PDF) [file pone.0235853.s004.pdf]

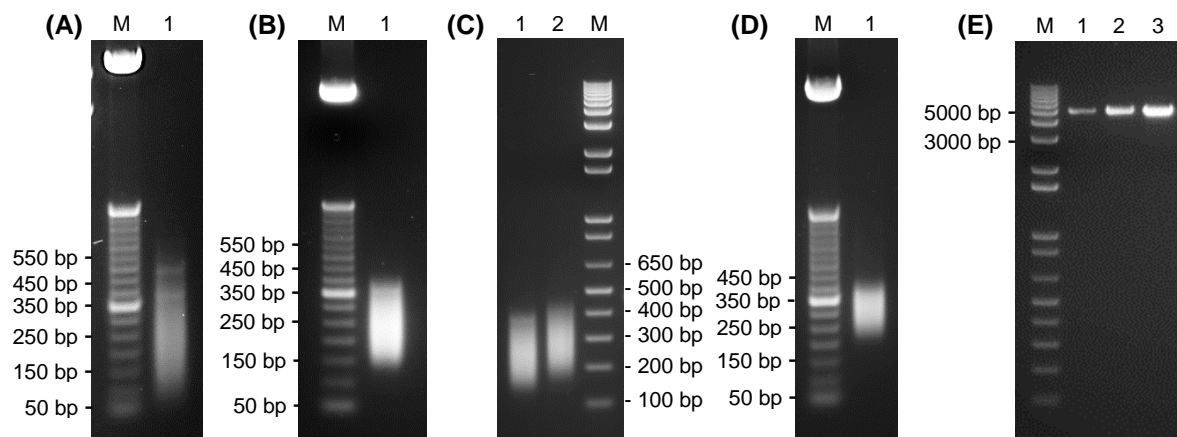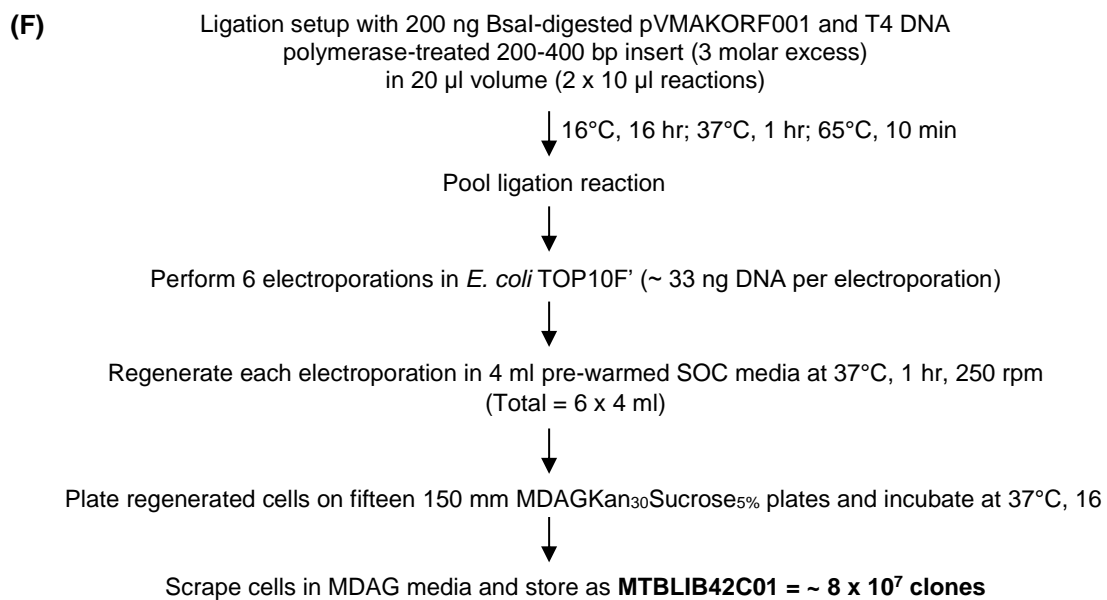

Supplement: S5 Fig — (A) Analysis of preparative sheared pool of 28 M. tuberculosis genes using agarose gel. The 28 genes (excluding CFP10 and ESAT-6) were pooled and sheared using Covaris ultrasonicator to obtain 100–500 bp fragments as per manufacturer’s instructions. Lane M, 50 bp DNA ladder; Lane 1, Sheared DNA pool. (B) Agarose gel-based size selection of the sheared DNA. Size selection of the sheared DNA was performed using 1.2% SYBR safe agarose gel to obtain fragment in the size range of 150–400 bp. Lane M, 50 bp DNA ladder; Lane 1, Size selected DNA. (C) Analysis of DNA fragments before and after adapter ligation. Lane M, 1 kb DNA ladder; Lane 1, before adapter ligation; Lane 2, after adapter ligation. (D) Analysis of T4 DNA polymerase-treated insert. Lane M, 50 bp DNA ladder; Lane 1, T4 DNA polymerase-treated 220–380 bp insert. (E) Analysis of BsaI-digested vector pVMAKORF001. M-1 kb DNA ladder, Lane 1, 2 and 3- Dilutions of the digested vector pVMAKORF001. (F) Workflow for the construction and storage of MTBLIB42C01 library. (PDF) [file pone.0235853.s005.pdf]

Fig 3

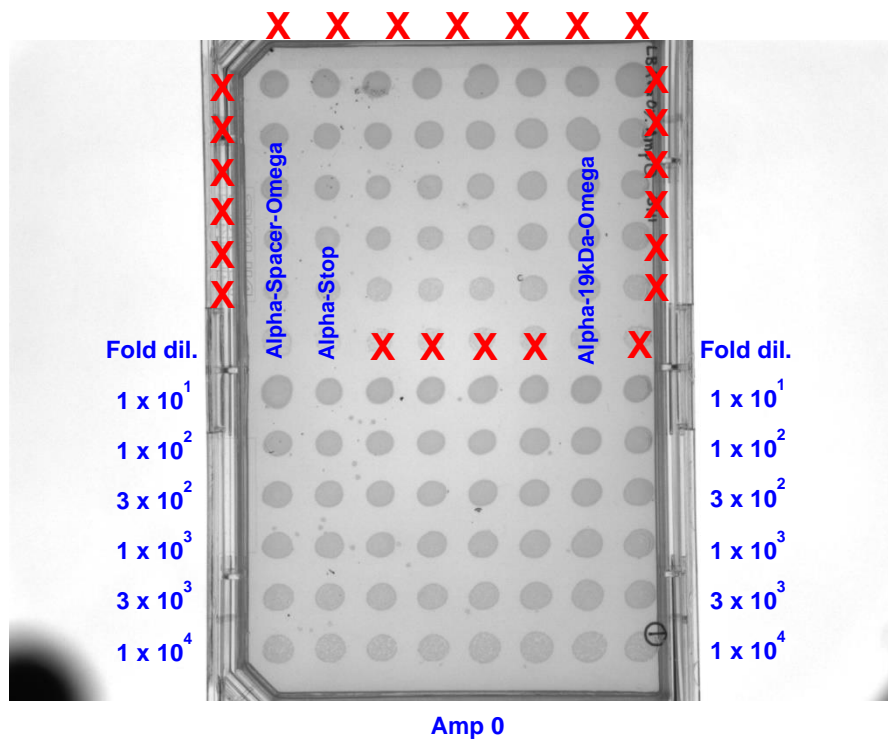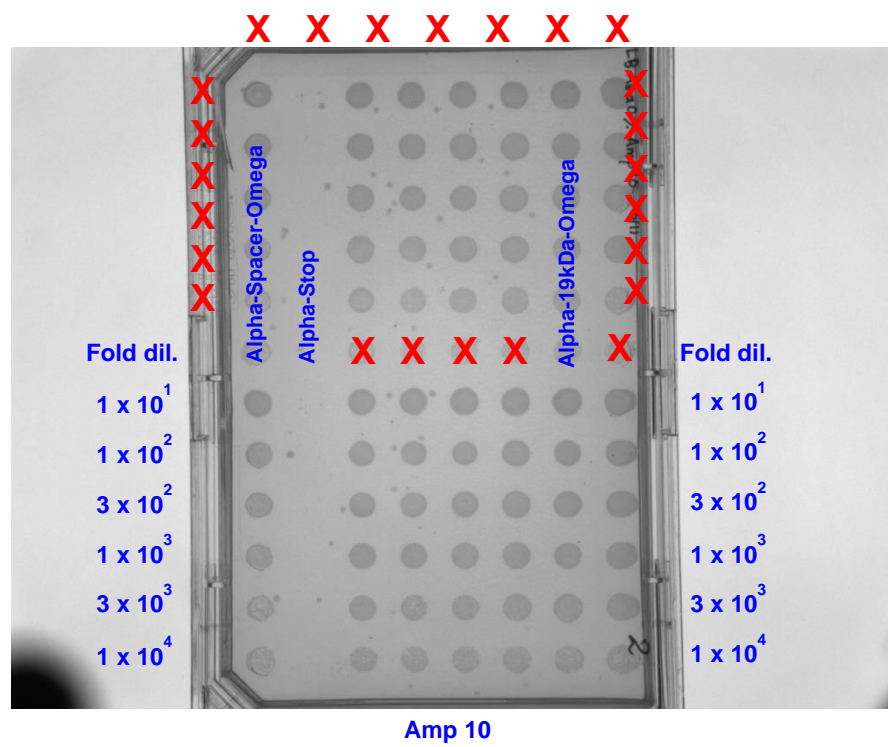

Fig 3

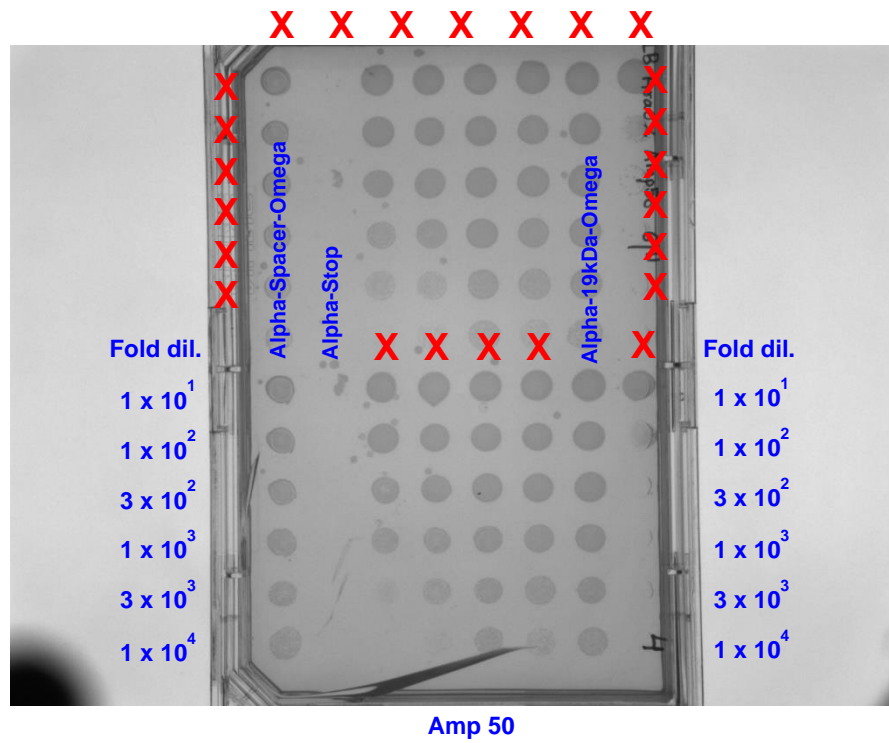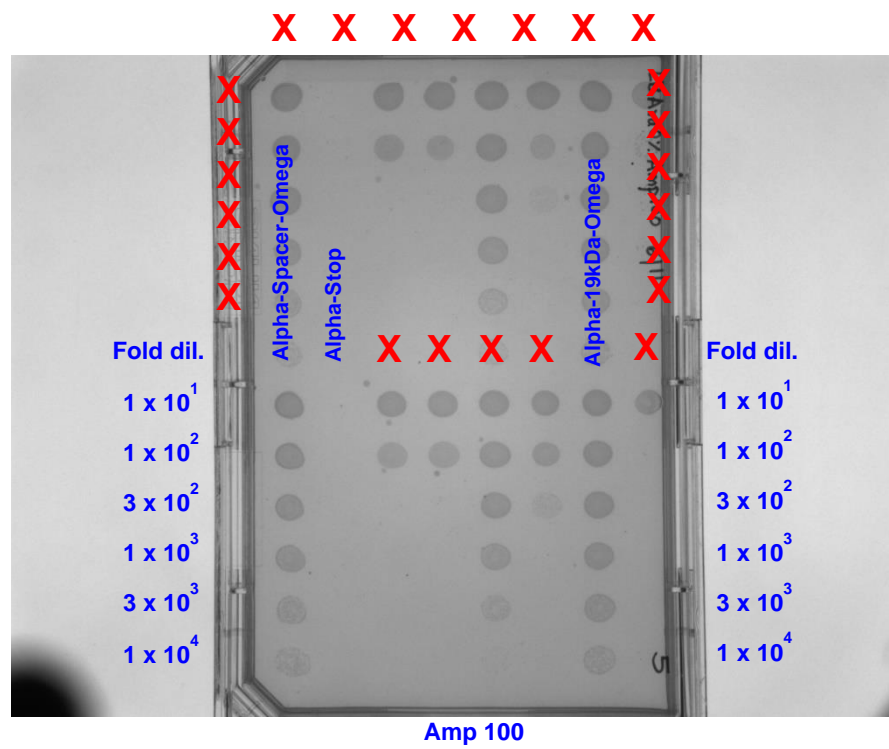

**Fig 5**

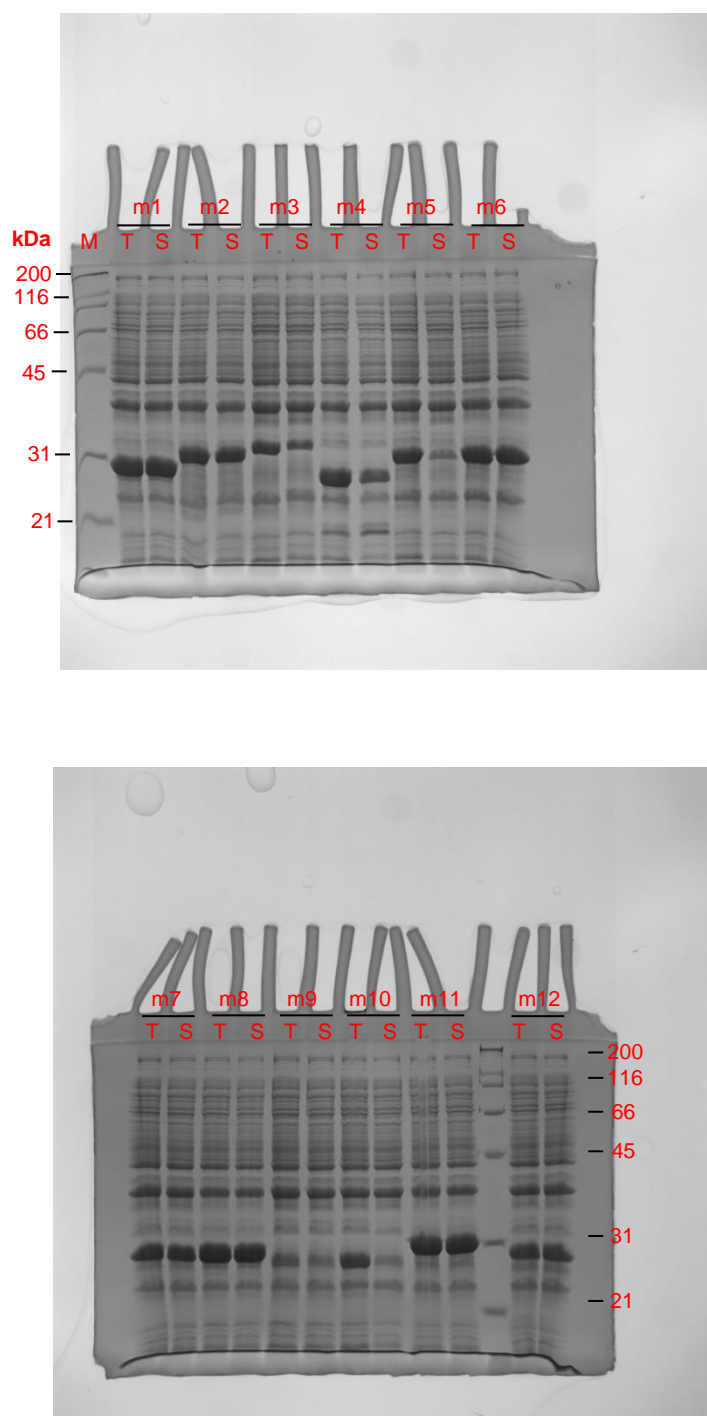

S1

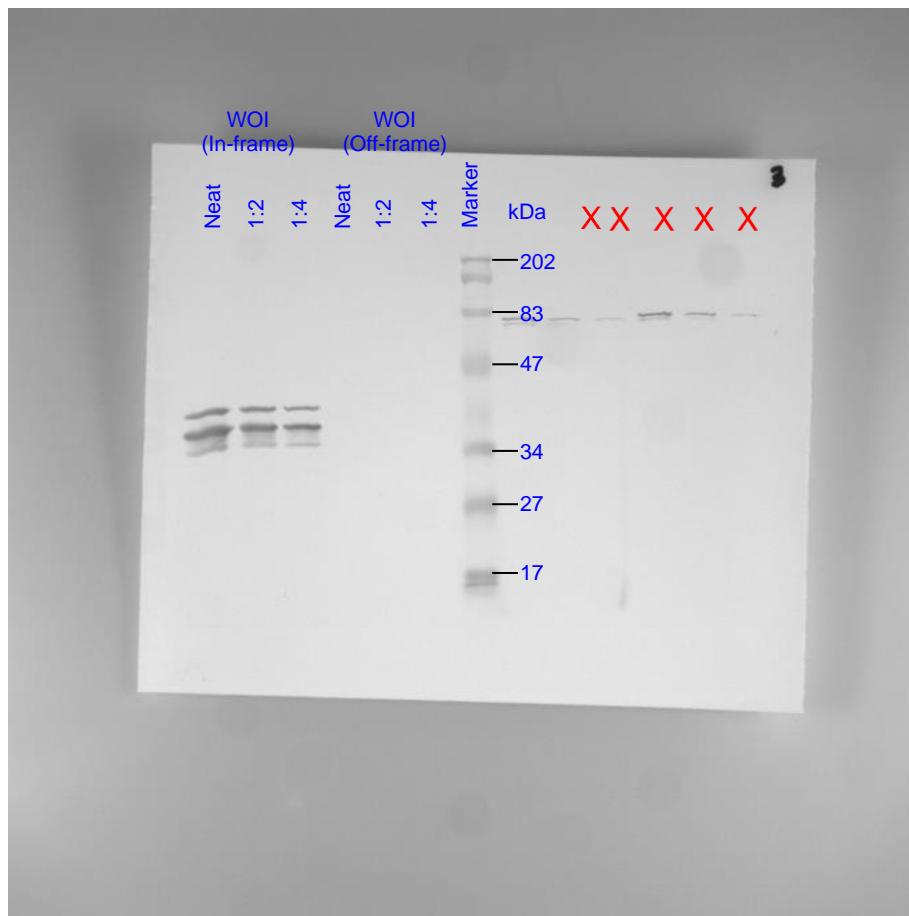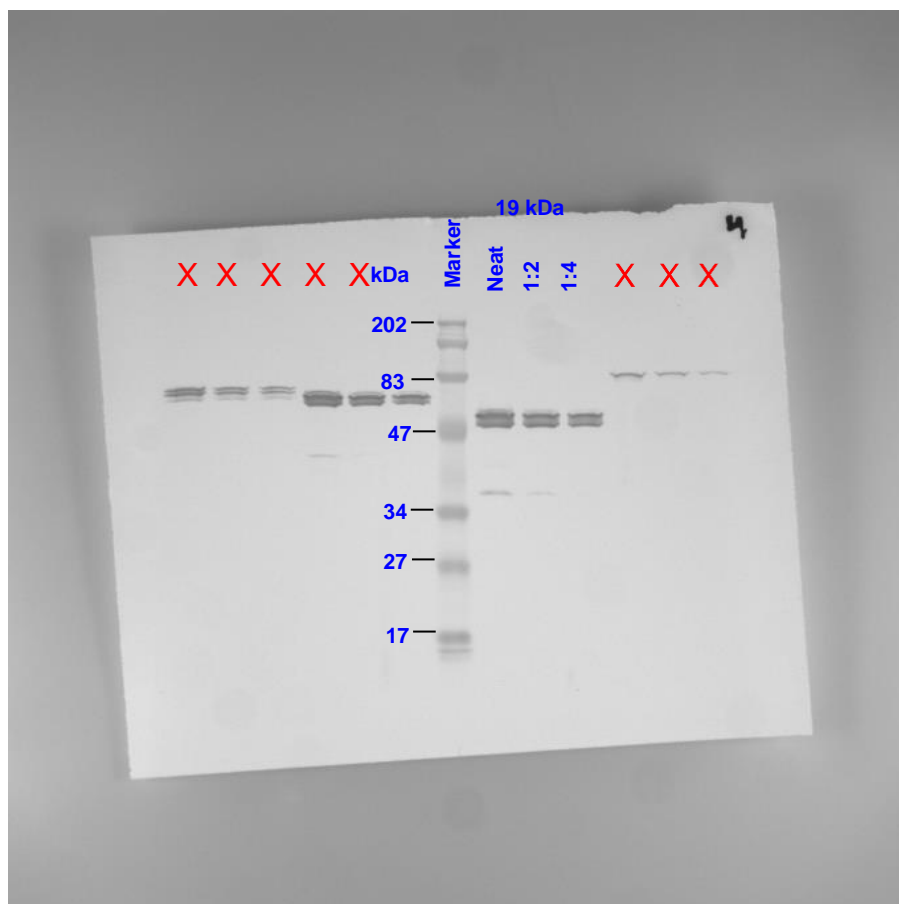

S2

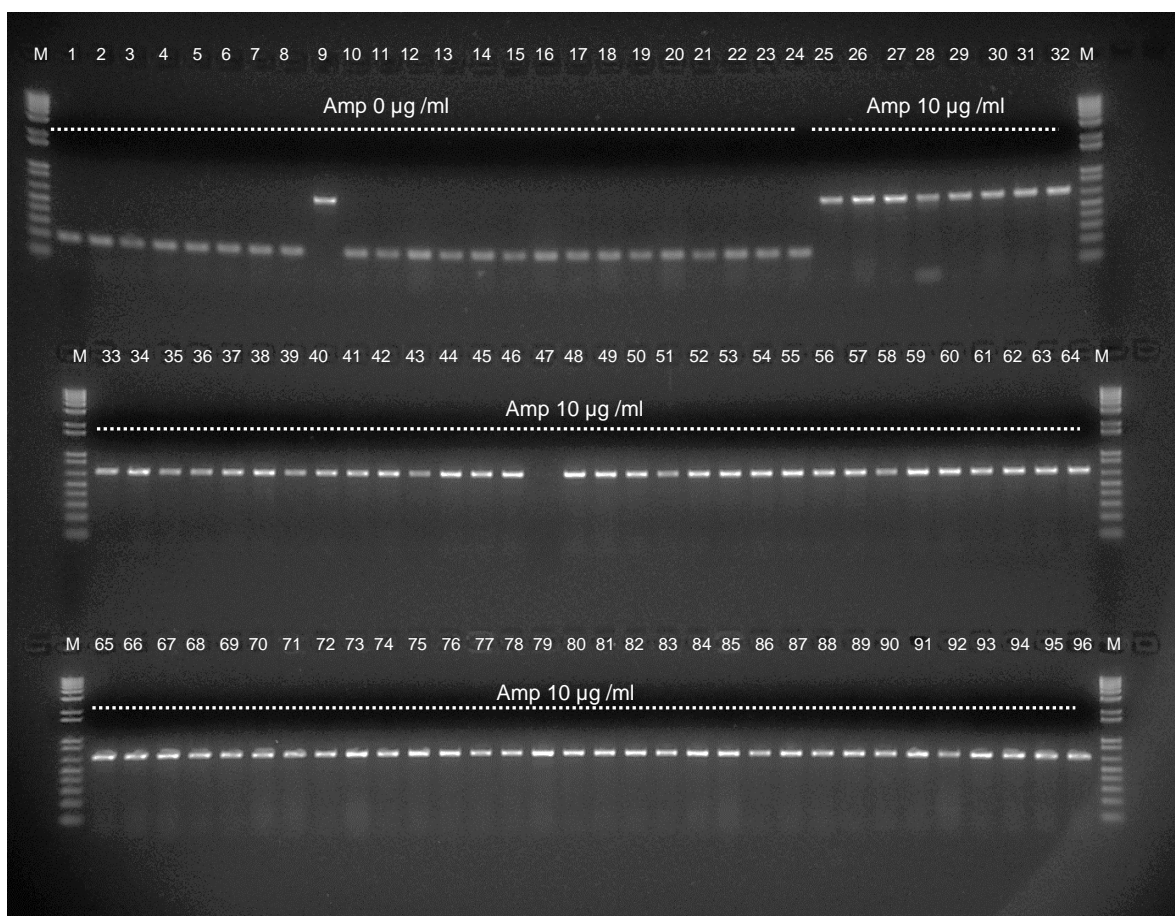

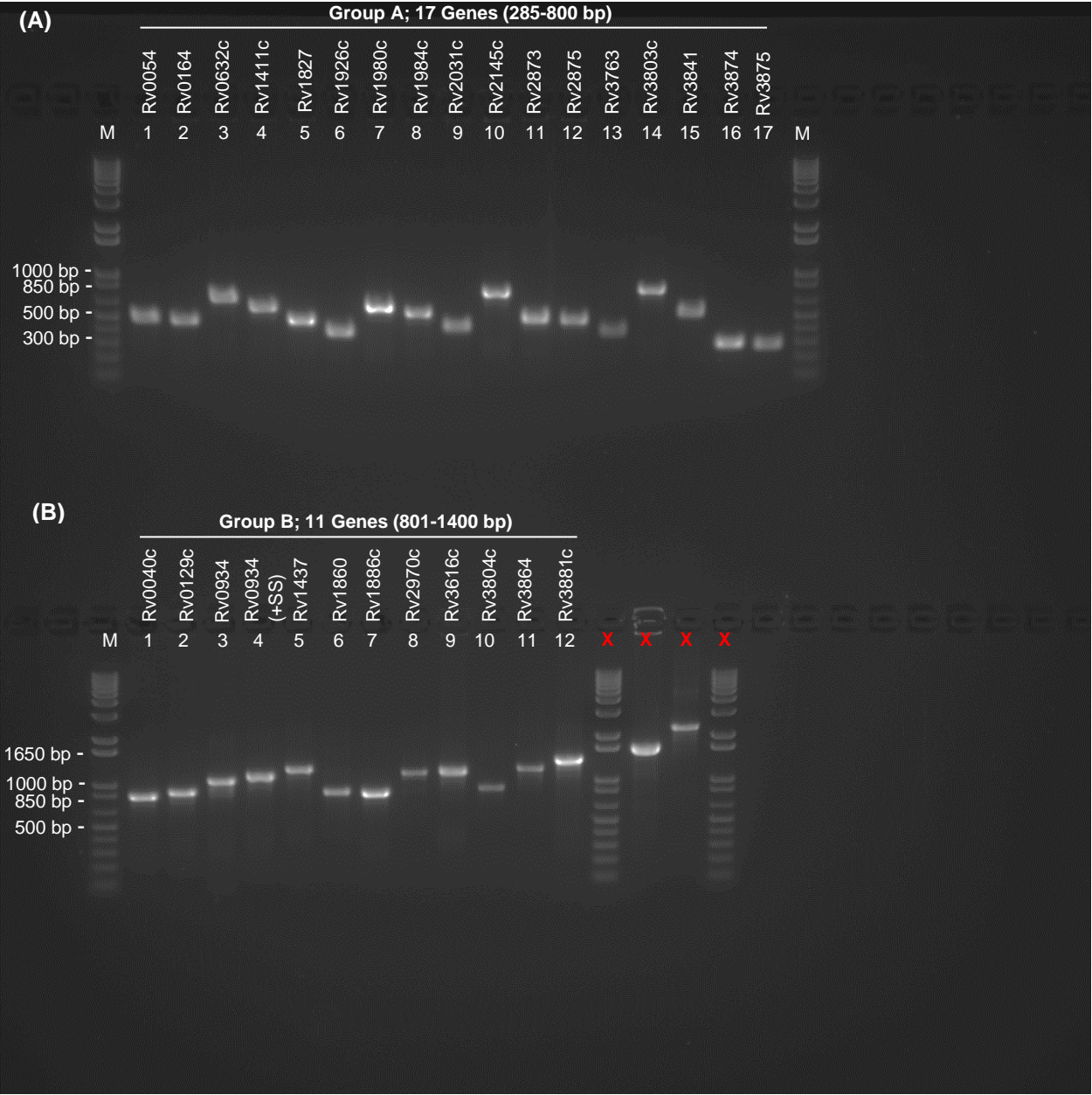

S4 Figure (C)

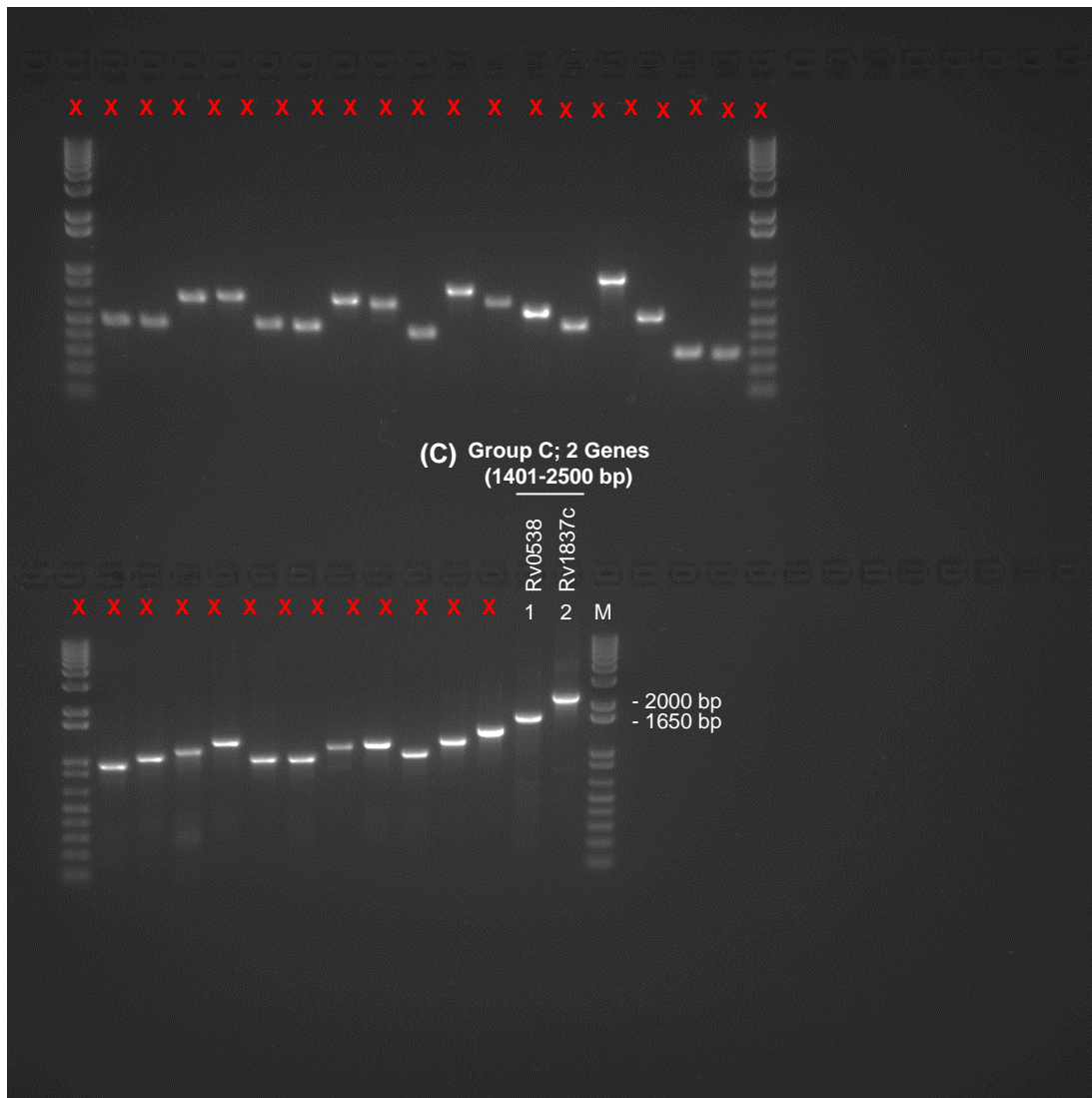

S5 Figure (A)

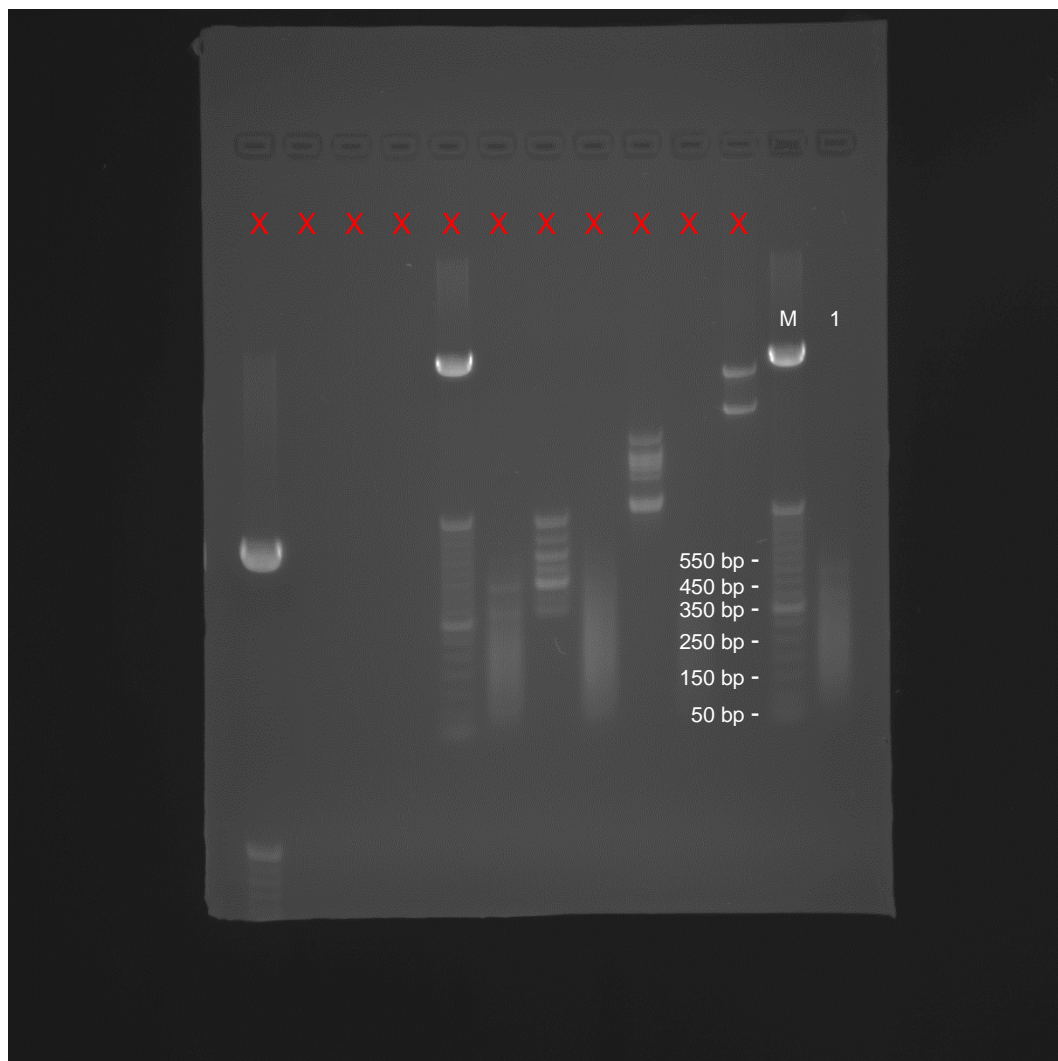

**S5 Figure (B)**

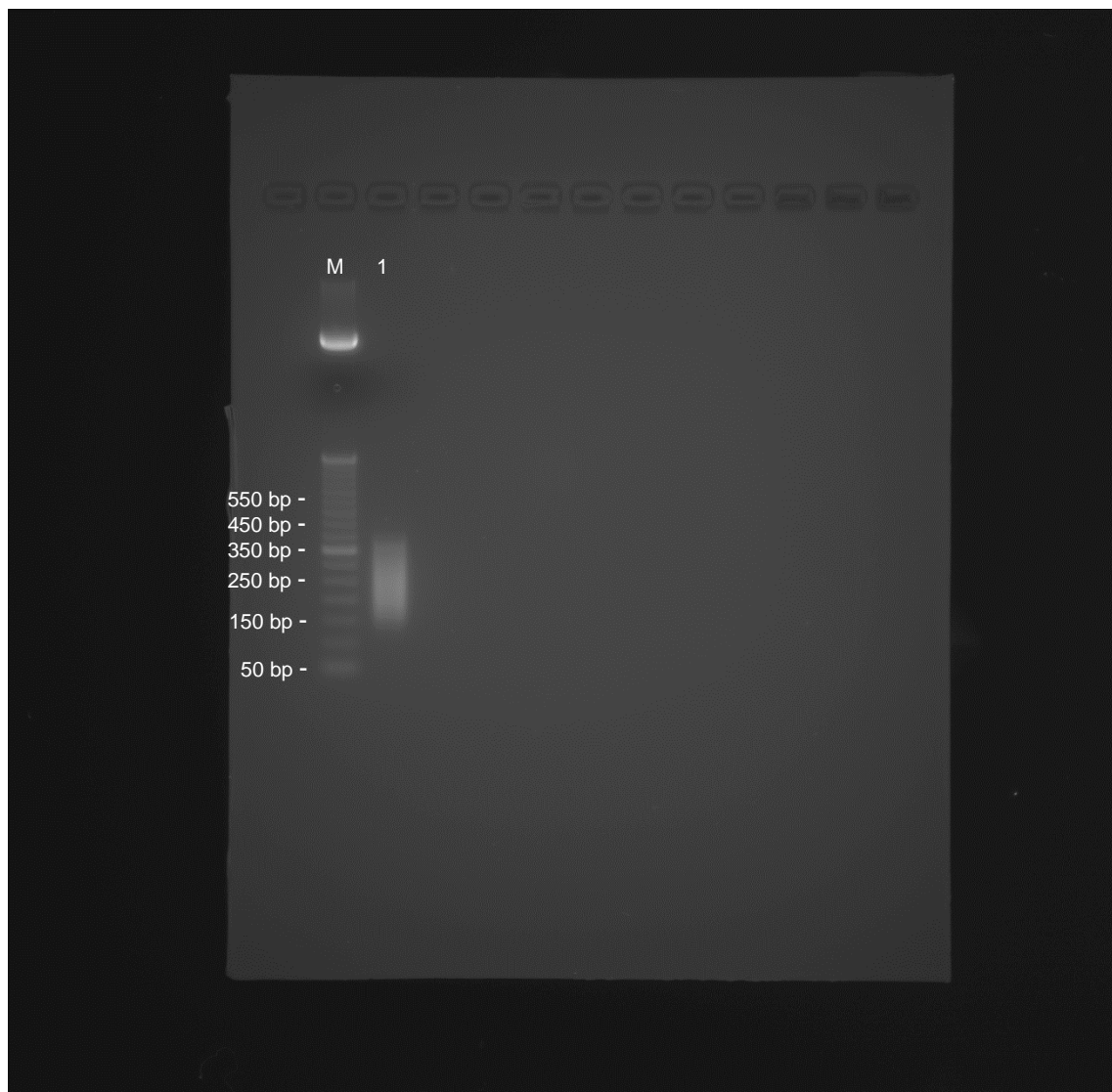

**S5 Figure (C)**

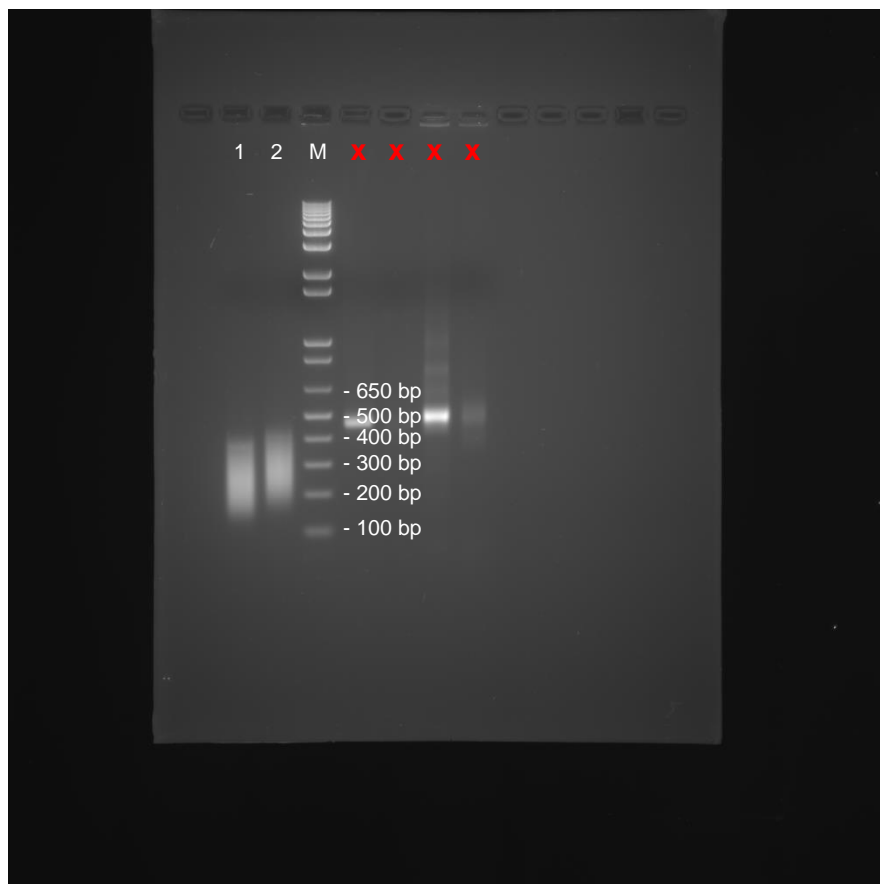

S5 Figure (D)

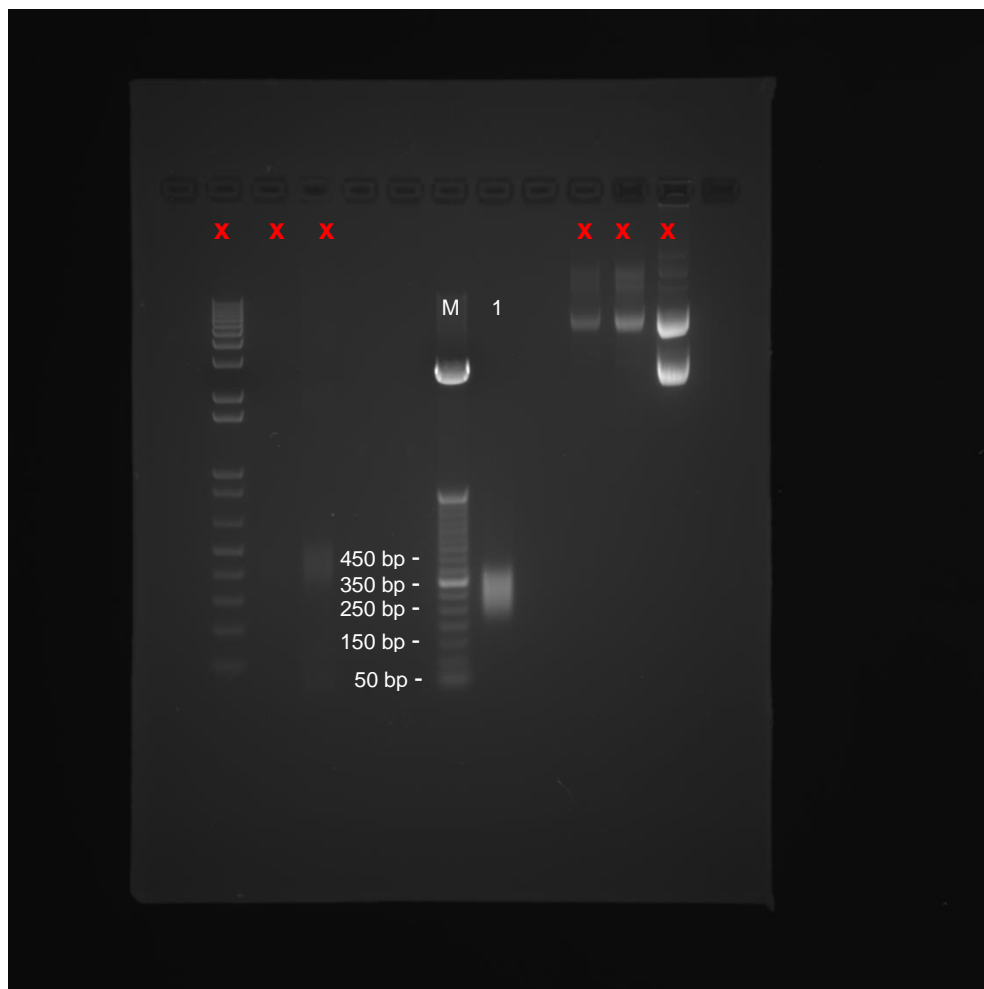

**S5 Figure (E)**

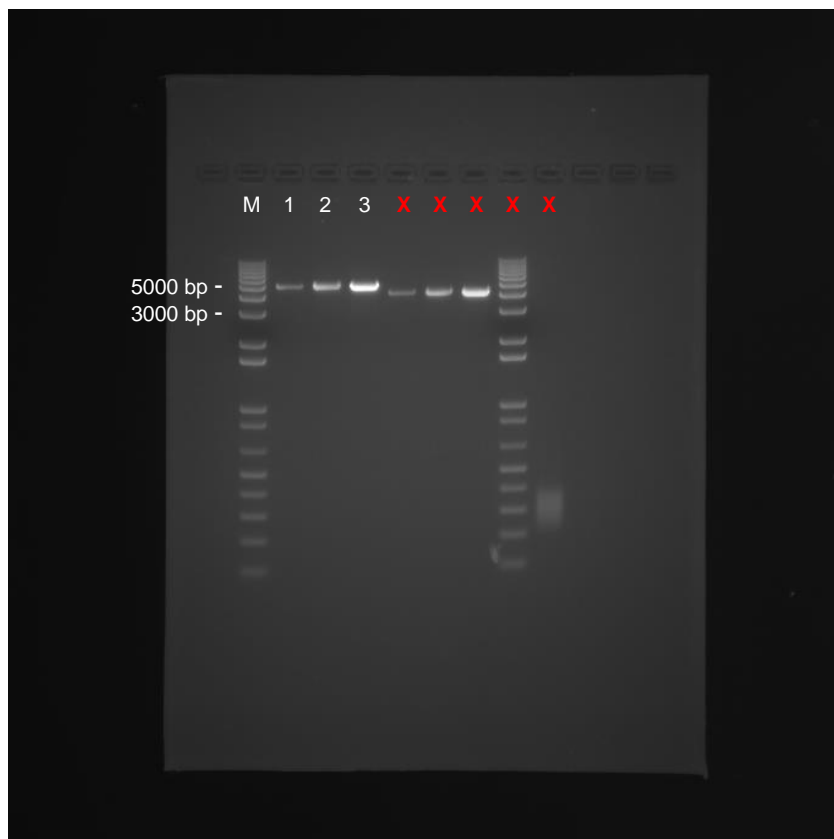

**S6 Figure (C and D)**

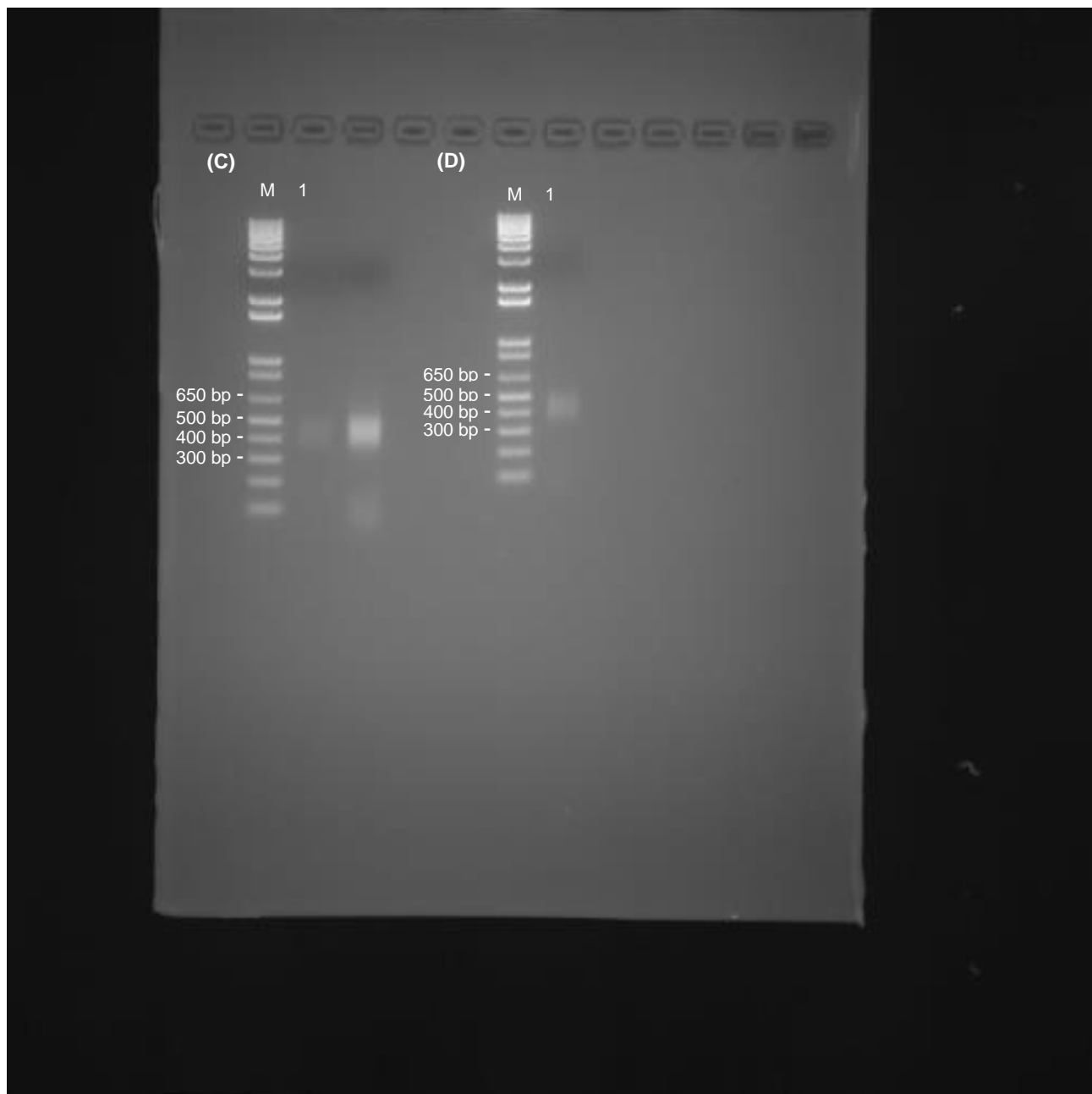

Supplement: S1 Raw images — (PDF) [file pone.0235853.s011.pdf]
