# Supplementary material for: An efficient ORF selection system for DNA fragment libraries based on split beta-lactamase complementation
Source: PLoS One. 2020 Jul 23;15(7):e0235853. doi: 10.1371/journal.pone.0235853 (PMC7377443; doi:10.1371/journal.pone.0235853)

(A)

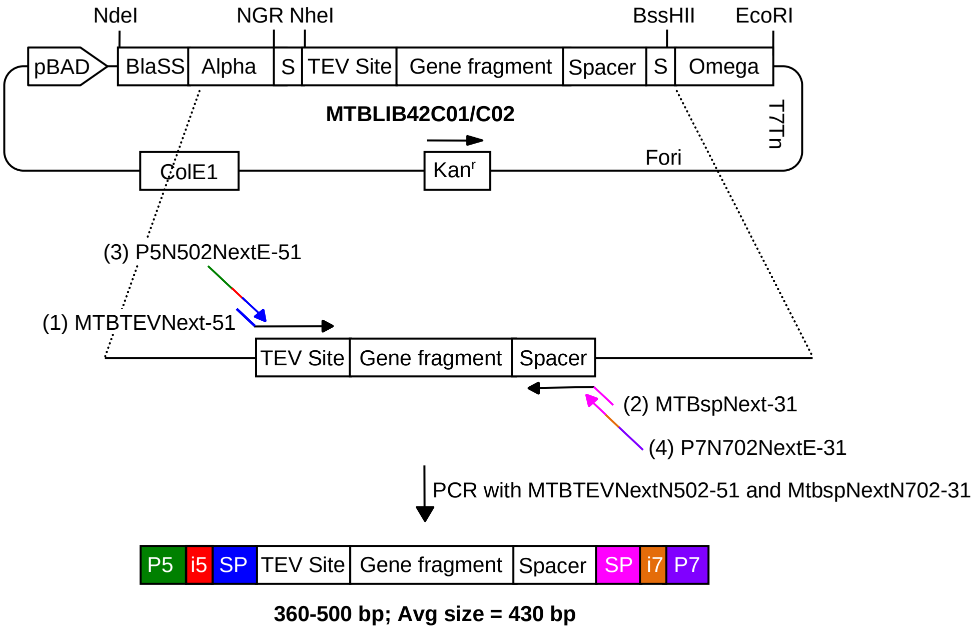

(B)

Primer	Sequence (5′-3′)
(1)MTBTEVNext51	NGS priming site Priming site<br>TCGTCGGCAGCGTCAGATGTGTATAAGAGACAGCGGCAGCGAAAATCTCTACTTCCAA*G<br>(33 base) (26 base)
(2)MTBSpNext31	NGS priming site Priming site<br>GTCTCGTGGGCTCGGAGATGTGTATAAGAGACAGCCACCGCCAGCACCTCCTGAAGCAC*C<br>(33 base) (26 base)
(3)P5N502NextE-51	Hybridisation site;P5 i5-N502<br>AATGATACGGCGACCACCGAGATCTACACCTCTCTATTCGTCGGCAGCGTCAGATGT*G<br>(29 base) (8 base) (21 base)
(4)P7N702NextE-31	Hybridisation site;P7 i5-N702 NGS priming site<br>CAAGCAGAAGACGGCATACGAGATCTAGTACGGTCTCGTGGGCTCGGAGATGTG*T<br>(29 base) (8 base) (23 base)

(C)

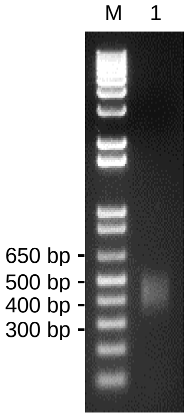

(D)

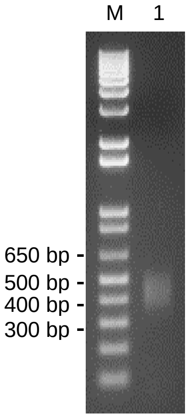

Supplement: S6 Fig — (A) PCR strategy based on 2 overlapping primers based on primers described by Illumina but with modified outer indexed primers for improved annealing to the inner primer. (B) Details of the primers employed during amplification. * indicates phosphorothioate bond. (C) Analysis of MTBLIB42C01 library amplified using emulsion PCR. Lane M, 1 kb DNA ladder; Lane 1, MTBLIB42C01 dual-indexed library. (D) Analysis of MTBLIB42C02 library amplified using emulsion PCR. Lane M, 1 kb DNA ladder; Lane 1, MTBLIB42C02 dual-indexed library (PDF) [file pone.0235853.s006.pdf]
