# Supplementary material for: An efficient ORF selection system for DNA fragment libraries based on split beta-lactamase complementation
Source: PLoS One. 2020 Jul 23;15(7):e0235853. doi: 10.1371/journal.pone.0235853 (PMC7377443; doi:10.1371/journal.pone.0235853)
Supplement: S1 Table — * denotes that these genes were purified from agarose gel, rest were purified using QIAquick PCR purification kit. NA denotes that these genes were not pooled in the gene mix subjected to shearing. # denotes that these genes were added as full-length genes in 10 μg 200–400 bp sheared DNA. (PDF) [file pone.0235853.s008.pdf]

| Group A (285-800 bp); 17 genes | | | | | |
|---|---|---|---|---|---|
| **S.No.** | **Rv no.** | **Name** | **Size (bp)** | **Amplification conditions** | **Amount for 7 pmoles (µg)** |
| 1 | Rv0054 | ssb | 492 | HF Pol, 2 % DMSO | 2.3 |
| 2 | Rv0164 | TB18.50 | 483 | HF Pol, 2 % DMSO | 2.2 |
| 3 | Rv0632c | Ech | 693 | HF Pol, 2 % DMSO | 3.2 |
| 4 | Rv1411c | lprG | 603 | HF Pol, 2 % DMSO | 2.8 |
| 5 | Rv1827 | garA | 486 | HF Pol, 2 % DMSO | 2.2 |
| 6 | Rv1926c | MPT63 | 390 | HF Pol, 2 % DMSO | 1.8 |
| 7 | Rv1980c | MPT64 | 615 | HF Pol, 2 % DMSO | 2.8 |
| 8 | Rv1984c | CFP21 | 555 | HF Pol, 2 % DMSO | 2.6 |
| 9 | Rv2031c | 14 kDa | 432 | HF Pol, 2 % DMSO | 2.0 |
| 10 | Rv2145c | Wag31 | 780 | HF Pol, 2 % DMSO | 3.6 |
| 11 | Rv2873 | MPT83 | 501 | HF Pol, 2 % DMSO | 2.3 |
| 12 | Rv2875 | Mpt70 | 489 | HF Pol, 2 % DMSO | 2.2 |
| 13 | Rv3763 | 19 kDa | 381 | HF Pol, 2 % DMSO* | 1.8 |
| 14 | Rv3803c | MPT51 | 798 | HF Pol, 2 % DMSO | 3.7 |
| 15 | Rv3841 | BfrB | 543 | HF Pol, 2 % DMSO* | 2.5 |
| 16 | Rv3874 | CFP10 | 300 | HF Pol, 2 % DMSO | NA (6 ng#) |
| 17 | Rv3875 | ESAT-6 | 285 | HF Pol, 2 % DMSO | NA (6 ng#) |
| Total (of 15 genes) | | | | | **37.9 µg** |
| **Group B (801-1400 bp); 11 genes** | | | | | |
| **S.No.** | **Rv no.** | **Name** | **Size (bp)** | **Amplification conditions** | **Amount for 3.5 pmoles (µg)** |
| 1 | Rv0040c | MTC28 | 834 | HF Pol, 2 % DMSO | 1.9 |
| 2 | Rv0129c | Ag85C | 882 | HF Pol, 2 % DMSO | 2.0 |
| 3 | Rv0934 | 38 kDa | 1122/1050 | HF Pol, 2 % DMSO | 1.3 ug each |
| 4 | Rv1437 | Pgk | 1236 | HF Pol, 2 % DMSO | 2.9 |
| 5 | Rv1860 | MPT32 | 858 | HF Pol, 2 % DMSO* | 2.0 |
| 6 | Rv1886c | Ag85B | 855 | HF Pol, 2 % DMSO | 2.0 |
| 7 | Rv2970c | LipN | 1128 | HF Pol, 2 % DMSO* | 2.6 |
| 8 | Rv3616c | EspA | 1176 | HF Pol, 2 % DMSO | 2.7 |
| 9 | Rv3804c | Ag85A | 885 | HF Pol, 2 % DMSO | 2.0 |
| 10 | Rv3864 | EspE | 1206 | HF Pol, 2 % DMSO* | 2.8 |
| 11 | Rv3881c | mtb48 | 1380 | HF Pol, 2 % DMSO | 3.2 |
| Total (11 genes) | | | | | **24.1 µg** |
| **Group C (1401-2500 bp); 2 genes** | | | | | |
| **S.No.** | **Rv no.** | **Name** | **Size (bp)** | **Amplification conditions** | **Amount for 3.5 pmoles (µg)** |
| 1 | Rv0538 | PTRP | 1644 | Pfu Pol, 4 % DMSO | 3.8 |
| 2 | Rv1837c | 81 kDa | 2223 | Pfu Pol, 6 % DMSO, 2 Step | 5.1 |
| Total (2 genes) | | | | | **8.9 µg** |
