# Supplementary material for: An efficient ORF selection system for DNA fragment libraries based on split beta-lactamase complementation
Source: PLoS One. 2020 Jul 23;15(7):e0235853. doi: 10.1371/journal.pone.0235853 (PMC7377443; doi:10.1371/journal.pone.0235853)
Supplement: S2 Table — The primers were designed for amplification of 30 genes without stop codon using M. tuberculosis genomic DNA as the template. Rv0934 (38 kDa) gene was amplified using 2 different 5’ primers with same 3’ primer to obtain gene with and without signal sequence. Rv1837c (81 kDa) was amplified using two step PCR. (PDF) [file pone.0235853.s009.pdf]

S. No.	Gene	Primer name	Length (bases)	Sequence (5' – 3')
1.	Rv0040c (MTC28)	Rv0040c-SS-51	27	GATCCCCTGCTTCCTCCACCGCCTATC
		Rv0040c-31	20	GCGCGGCGGGACCGGTGTCA
2.	Rv0054 (ssb)	Rv0054-51	22	GTGGCTGGCGACACCACCATCA
		Rv0054-31	19	GAATGGTGGTTCGTCATCG
3.	Rv0129c (Ag85C)	Rv0129c-SS-51	24	TTCTCTAGACCCGGTCTTCCAGTG
		Rv0129c-31	22	GGCGGCAGGGGCAGCAGGGGCG
4.	Rv0164 (TB18.50)	Rv0164-51	23	ATGACCGCAATCTCGTGCTCACC
		Rv0164-31	21	ACTCGCGGCAAGCTGCTCGGC
5.	Rv0538 (PTRP)	Rv0538-51	22	ATGGATGTCGCTTTGGGGGTTG
		Rv0538-31	21	GAAGCCGCCAAATAAGTCGCC
6.	Rv0632c (ECH)	Rv0632c-51	24	ATGAGTGATCCGGTCAGCTATACC
		Rv0632c-31	23	AAGACCAAACTCGGCTGCTATCC
7.	Rv0934 (38 kDa) 51+32 = -SS 52+32 = +SS	Rv0934-SS-51	26	GGTTCTAAGCCACCCAGCGGTTCGCC
		Rv0934-SS-52	25	GTGAAAATTCGGTTGCATACGCTGT
		Rv0934-32	24	GCTGGAAATCGTAGCAATCAACGC
8.	Rv1411c (lpr)	Rv1411c-SS-51	25	GGACCACTACCTGACGCAAAGCCGC
		Rv1411c-31	22	GCTAACAGGAGGCTTCGTGACC
9.	Rv1437 (pgk)	Rv1437-51	29	ATGTCCGTGGCAAACCTCAAGGATCTACT
		Rv1437-31	22	CAAAACTCCTCCGGTTGGCTGC
10.	Rv1827 (garA)	Rv1827-51	26	GTGACTGACATGAACCCGGATATTGA
		Rv1827-31	28	TGGTCCTCCCGTACTCCCGTCATCCTCG
11.	Rv1837c (81 kDa) Step 1 PCR	Mtb81-51	23	TGCCAGTAAGGGAGGAAGCAATG
		Mtb81-32	35	CACAGCGGACGCGTTACGGGCCGCATCGTCACCGG
	Step 2 PCR	Rv1837c-51	22	ATGACGGATCGCGTGTCGGTGG
		Rv1837c-31	25	GCGAGCTGCATCGTCTCCGGCCCTG
12.	Rv1860 (MPT32)	Rv1860-SS-51	24	GATCCAGAGCCTGCGCCCCCGGTA
		Rv1860-31	24	GGCCGGTAAGGTACGCTGCGGTGT
13.	Rv1886c (Ag85B)	Rv1886c-SS-51	25	TTCTCCCGTCCAGGGCTGCCGGTCG
		Rv1886c-31	22	GCCTGCGCCTAACGAACTCTGC
14.	Rv1926c (MPT63)	Rv1926c-SS-51	23	GCCTATCCCATCACCGGAAAACT
		Rv1926c-31	24	CGGCTCCCAAATCAGCAGATCCTC
15.	Rv1980c (MPT64)	Rv1980c-SS-51	24	GCGCCCAAGACCTACTGCGAGGAG
		Rv1980c-31	24	GGCAAGCATCGAGTCAATCGCGGA
16.	Rv1984c (CFP21)	Rv1984c-SS-51	25	GATCCGTGTTCGGACATTGCGGTCG
		Rv1984c-31	24	TCCGGCATGGTCCAGCCTGTTCGC
17.	Rv2031c (14 kDa)	Rv2031c-51	21	ATGGCGACCACCCTTCCCGTT
		Rv2031c-31	22	GTTGGTGGACCGGATCTGAATG
18.	Rv2145c (Wag31)	Rv2145c-51	21	ATGCCGCTTACACCTGCCGAC
		Rv2145c-31	22	GTTTTTGCCCCGGTTGAATTGA
19.	Rv2873 (MPT83)	Rv2873-SS-51	23	GACCCGGCAGCAGACCTGATTGG
		Rv2873-31	24	CTGCGCGGGGGGCATCAGCACCGT
20.	Rv2875 (MPT70)	Rv2875-SS-51	24	GGCGATCTGGTGGGTCCTGGCTGC
		Rv2875-31	24	CGCCGGAGGCATTAGCACGCTGTC

21.	Rv2970c (LipN)	Rv2970c-51	23	ATGACGAAGAGTCTGCCAGGTGT
		Rv2970c-31	25	AACACGGCTAAGGTGGGCACGCAGC
22.	Rv3616c (EspA)	Rv3616c-51	27	ATGAGCAGAGCGTTTATCATAGATCCA
		Rv3616c-31	21	GACCACGTTTCGTACCAGCAC
23.	Rv3763 (19 kDa)	Rv3763-SS-51	23	GAGACCACGACAGCGGCAGGCAC
		Rv3763-31	21	GGAACACGTCACCTCGATTTC
24.	Rv3803c (MPT51)	Rv3803c-SS-51	24	GCCCCTTACGAGAACCTGATGGTG
		Rv3803c-32	24	GCGGATCGCTCCGACGATATCGCC
25.	Rv3804c (Ag85A)	Rv3804c-SS-51	25	TTTTCTCGTCCTGGCTTGCCGGTGG
		Rv3804c-31	25	GGCTCCTTGTGGCGCAGGCCCGGTG
26.	Rv3841 (BfrB)	Rv3841-51	23	ATGACAGAATACGAGGGGCCTAA
		Rv3841-31	21	GAGGCGACCCCCAGCAGCGTG
27.	Rv3864 (EspE)	Rv3864-51	24	ATGGCGTCGGGTAGCGGTCTTTGC
		Rv3864-31	27	AAGCACAGTCCCCTCCTGCCCCCGTTG
28.	Rv3874 (CFP10)	Rv3874-51	23	ATGGCGGAGATGAAGACCGATGC
		Rv3874-31	23	GAAACCCATTTGCGAGGACAGCG
29.	Rv3875 (ESAT-6)	Rv3875-51	20	ATGACAGAGCAGCAGTGGAA
		Rv3875-31	23	TGCGAACATCCCAGTGACGTTGC
30.	Rv3881c (MTB48)	Rv3881c-51	23	ATGACGCAATCGCAGACCGTGAC
		Rv3881c-31	23	CTTCGATTCCTTACTGTCCTGGC
