# Supplementary material for: An efficient ORF selection system for DNA fragment libraries based on split beta-lactamase complementation
Source: PLoS One. 2020 Jul 23;15(7):e0235853. doi: 10.1371/journal.pone.0235853 (PMC7377443; doi:10.1371/journal.pone.0235853)
Supplement: S3 Table — Bases in blue indicate the additional 7-bases required to generate 4 base 5’ BsaI compatible overhangs after T4 DNA Polymerase treatment in presence of dTTP. Bio denotes Biotin moiety attached to the 5’ end of the primer Bio L1-s. * denotes one phosphorothioate bond present at the 3’-end of both the amplification primers. The amino acid sequence encoded by the template to which primers anneal is shown in bold. (PDF) [file pone.0235853.s010.pdf]

S.No.	Primer	Role	Length	Sequence (5’ – 3’)
1.	Bio L1-s	Adapter duplex; sense strand	34 mer	5′ Bio-CGGCAGCGAAAATCTCTACTTCCAAGGAGCATC*T 3′
2.	L2	Adapter duplex; anti-sense strand	29 mer	5′ GATGCTCCTTGGAAGTAGAGATTTTCGCT -3′
3.	**5’ Adapter sequence (Adapter L)**			**G S E N L Y F Q G A S**<br>5′ Bio-CGGCAGCGAAAATCTCTACTTCCAAGGAGCATC*T<br>3′ TCGCTTTTAGAGATGAAGGTTCCTCGTAG
4.	L3-s	5’ primer for DNA amplification	34 mer	5′ CGGCAGCGAAAATCTCTACTTCCAAGGAGCATC*T 3′
5.	K1	Adapter duplex; sense strand	26 mer	5′ GTGGTGCTTCAGGAGGTGCTGGCGGT 3′
6.	K2-s	Adapter duplex; anti-sense strand, 3’ primer for DNA amplification	31 mer	5′ CTCCACCGCCAGCACCTCCTGAAGCACCAC*T 3′
7.	**3’ Adapter sequence (Adapter K)**			**S G A S G G A G G**<br>5′ GTGGTGCTTCAGGAGGTGCTGGCGGT 3′<br>3′ T*CACCACGAAGTCCTCCACGACCGCCACCTC 5′
