# Supplementary material for: An efficient ORF selection system for DNA fragment libraries based on split beta-lactamase complementation
Source: PLoS One. 2020 Jul 23;15(7):e0235853. doi: 10.1371/journal.pone.0235853 (PMC7377443; doi:10.1371/journal.pone.0235853)
Supplement: S7 Fig — Only relevant genes and restriction sites are shown. The maps are not to scale. Ampr, ampicillin resistance marker; ColE1, origin of replication; Fori, phage M13 origin of replication. RBS, Ribosome binding site; T7PO, T7 promoter-operator; H10, Deca-histidine tag; D, Bacteriophage Lambda coat protein D; TEV, Tobacco etch virus protease cleavage site; SacB, 2 kb B. subtilis levansucrase gene; BAP, Biotin Acceptor Peptide tag; S, spacer; GOI, Gene of interest. Sp, Glycine-serine rich spacer. (A) Gene fragments (GOI) appended by DNA sequence encoding TEV protease site (TEV) and spacer sequence (Sp) at 5’ and 3’ ends, respectively, were amplified using emulsion PCR from MTBLIB42C02, treated with T4 DNA polymerase in the presence of dTTP, and cloned into BsaI-digested pVMH10D-BAP001 vector. (B)-(C) Sequence of different components of pVMH10D-BAP001 expression vector (as denoted in the map). (PDF) [file pone.0235853.s007.pdf]

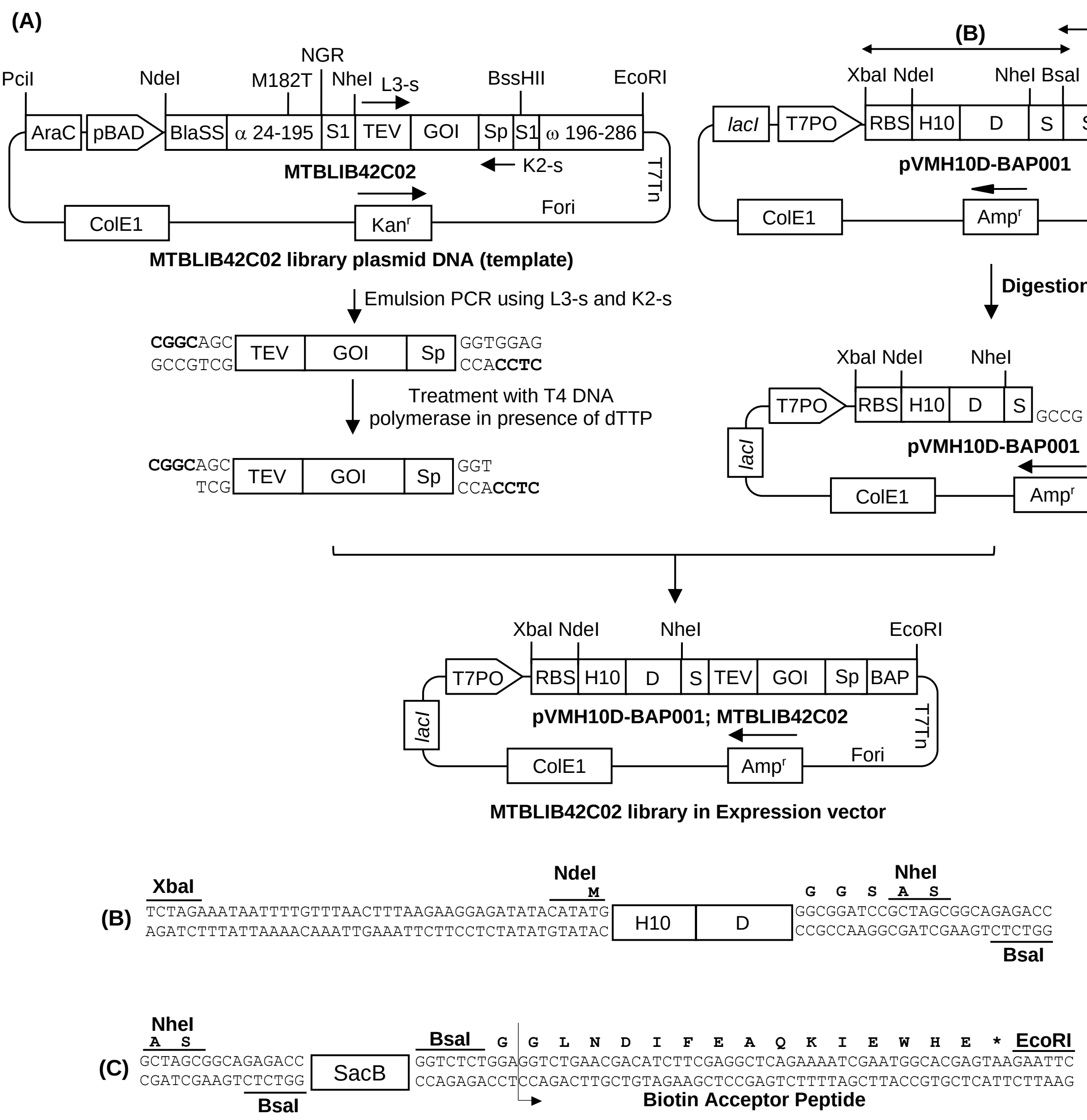
(A)
PciI
NdeI
M182T
NGR
NheI
L3-s
BssHII
EcoRI
AraC
pBAD
BlaSS
α 24-195
S1
TEV
GOI
Sp
S1
ω 196-286
MTBLIB42C02
K2-s
T7Tn
ColE1
Kanr
Fori
MTBLIB42C02 library plasmid DNA (template)
Emulsion PCR using L3-s and K2-s
CGGCAGC
GCCGTCG
TEV
GOI
Sp
GGTGGAG
CCACCTC
Treatment with T4 DNA polymerase in presence of dTTP
CGGCAGC
TCG
TEV
GOI
Sp
GGT
CCACCTC
(B)
(C)
XbaI
NdeI
NheI
BsaI
BsaI
EcoRI
lacI
T7PO
RBS
H10
D
S
SacB
BAP
pVMH10D-BAP001
Ampr
ColE1
Fori
Digestion with BsaI
XbaI
NdeI
NheI
EcoRI
T7PO
RBS
H10
D
S
GCCG
GGAG
BAP
lacI
pVMH10D-BAP001
T7Tn
ColE1
Ampr
Fori
XbaI
NdeI
NheI
EcoRI
T7PO
RBS
H10
D
S
TEV
GOI
Sp
BAP
lacI
pVMH10D-BAP001; MTBLIB42C02
T7Tn
ColE1
Ampr
Fori
MTBLIB42C02 library in Expression vector
(B)
XbaI
NdeI
M
TCTAGAAATAATTTTGTTTAACTTTAAGAAGGAGATATACATATG
AGATCTTTATTAAAACAAATTGAAATTCTTCCTCTATATGTATAC
H10
D
NheI
G G S A S
GGCGGATCCGCTAGCGGCAGAGACC
CCGCCAAGGCGATCGAAGTCTCTGG
BsaI
(C)
NheI
A S
GCTAGCGGCAGAGACC
CGATCGAAGTCTCTGG
BsaI
SacB
BsaI
G G L N D I F E A Q K I E W H E * EcoRI
GGTCTCTGGAGGTCTGAACGACATCTTCGAGGCTCAGAAAATCGAATGGCACGAGTAAGAATTC
CCAGAGACCTCCAGACTTGCTGTAGAAGCTCCGAGTCTTTTAGCTTACCGTGCTCATTCTTAAG
Biotin Acceptor Peptide
